# Leptotene/Zygotene Chromosome Movement Via the SUN/KASH Protein Bridge in *Caenorhabditis elegans*

**DOI:** 10.1371/journal.pgen.1001219

**Published:** 2010-11-24

**Authors:** Antoine Baudrimont, Alexandra Penkner, Alexander Woglar, Thomas Machacek, Christina Wegrostek, Jiradet Gloggnitzer, Alexandra Fridkin, Franz Klein, Yosef Gruenbaum, Pawel Pasierbek, Verena Jantsch

**Affiliations:** 1 Department of Chromosome Biology, Max F. Perutz Laboratories, University of Vienna, Vienna, Austria; 2 Department of Genetics, Hebrew University, Jerusalem, Israel; 3 Institute of Molecular Biotechnology of the Austrian Academy of Sciences, Vienna, Austria; 4 Institute of Molecular Pathology, Vienna, Austria; Harvard University, United States of America

## Abstract

The *Caenorhabditis elegans* inner nuclear envelope protein matefin/SUN-1 plays a conserved, pivotal role in the process of genome haploidization. CHK-2–dependent phosphorylation of SUN-1 regulates homologous chromosome pairing and interhomolog recombination in *Caenorhabditis elegans*. Using time-lapse microscopy, we characterized the movement of matefin/SUN-1::GFP aggregates (the equivalent of chromosomal attachment plaques) and showed that the dynamics of matefin/SUN-1 aggregates remained unchanged throughout leptonene/zygotene, despite the progression of pairing. Movement of SUN-1 aggregates correlated with chromatin polarization. We also analyzed the requirements for the formation of movement-competent matefin/SUN-1 aggregates in the context of chromosome structure and found that chromosome axes were required to produce wild-type numbers of attachment plaques. Abrogation of synapsis led to a deceleration of SUN-1 aggregate movement. Analysis of matefin/SUN-1 in a double-strand break deficient mutant revealed that repair intermediates influenced matefin/SUN-1 aggregate dynamics. Investigation of movement in meiotic regulator mutants substantiated that proper orchestration of the meiotic program and effective repair of DNA double-strand breaks were necessary for the wild-type behavior of matefin/SUN-1 aggregates.

## Author Summary

During meiosis, homologous chromosomes from each parent must pair, synapse, and recombine before being assorted to the gametes. In *Caenorhabditis elegans*, to find the correct pairing partner, telomere-led chromosome movement occurs in a restricted subvolume of the nucleus. This feature is comparable to the widely conserved meiotic bouquet, a configuration where telomeres cluster in a limited area at the nuclear periphery. Chromosomes are moved by cytoskeletal forces transmitted via the SUN/KASH bridge across the nuclear envelope, and abrogation of movement leads to precocious nonhomologous synapsis. Using live cell imaging, we followed the movement of matefin/SUN-1 aggregates, which highlight chromosome ends. Instead of single chromosome ends looking for their homologous partners, we observed that duplets/multiplets and single chromosome ends were brought together into “patches” by the ongoing movement during the leptotene/zygotene stages of meiosis. Chromosome ends then shuffled through these patches in search of the correct partner. This study was a comprehensive analysis of matefin/SUN-1 aggregate dynamics in wild type, known *Caenorhabditis elegans* pairing mutants, and the recombination mutant *spo-11*; and it examined the contributions of these genotypes to leptotene/zygotene chromosome movement.

## Introduction

During the first meiotic division, homologous parental chromosomes must accomplish numerous tasks that eventually result in their connection via homologous recombination. They must recognize one another, align, synapse via the tripartite proteinaceous synaptonemal complex (SC), and repair programmed double-strand breaks (DSBs); a subset of DSBs is repaired using the homologous partner as a template [1]. During this period, the chromosomes are connected to the nuclear envelope at one or both ends [2]. The highly conserved protein interaction module of SUN/KASH domain proteins has emerged as a core element for the attachment of chromosomal ends to the nuclear envelope, and for telomere-led chromosomal movement. The mechanism for moving chromosomes during early prophase I inside the nucleus via the SUN/KASH bridge, which provides a connection to various cytoskeletal forces in the cytoplasm, appears to be a general, evolutionarily conserved phenomenon (for reviews see [3]–[5]). Studies in *Saccharomyces cerevisiae* [6], [7], *Schizosaccharomyces pombe* [8], and maize [9] discovered differences among organisms with regard to which factors are employed to build the connection of the chromosome ends to the SUN-domain proteins in the inner nuclear envelope, and which cytoskeletal forces drive the movement. For example, in *S. cerevisiae*, telomere-led chromosome movement has been observed during meiotic prophase I, from leptotene to pachytene [6], [7], [10]. Interference with prophase chromosome movement in *S. cerevisiae* results in delayed pairing and DSB processing, aberrant crossover formation, and loss of crossover interference [6], [7], [11]–[16].

In many organisms, formation of the synaptonemal complex requires the formation of programmed meiotic DSBs; however, in *C. elegans*, synapsis is independent of DSBs [17]. In *C. elegans*, the SC is comprised of the lateral element components HTP-1 to 3 and HIM-3, and the central region components SYP-1 to SYP-4 [18], [19]. HTP-1, in addition to being part of the lateral element, also plays a role in licensing synapsis [20], [21].

Another characteristic of *C. elegans* is that the pairing of homologs involves homolog recognition regions (HRRs), also called pairing center (PC) regions, which are enriched in heterochromatic repeats localized at one end of each chromosome. HRRs were shown to be required to initiate the subsequent key features of meiosis I, recombination and disjunction [22]–[24]. The PC proteins ZIM-1 to ZIM-3 and HIM-8 bind to HRRs, and specifically localize to either one or two chromosomes [25]–[27]. When extrachromosomal arrays of the heterochromatic repeats found in HRRs are introduced into *C. elegans* germline cells, they recruit PC proteins and the arrays localize to the nuclear periphery [27].

In *C. elegans*, the protein matefin/SUN-1 (referred to as SUN-1 from this point forward) and its interacting partner ZYG-12 bridge the nuclear membranes and play a central role in the pairing of homologous chromosomes and the licensing of synapsis [28], [29]. The PC proteins colocalize with SUN-1, and are thought to connect chromosome ends to SUN-1 [28], [29]. A point mutation in the SUN domain of *C. elegans* SUN-1 revealed that prophase movement is required for chromosomes to find each other and to prevent nonhomologous synapsis [29], [30]. Recently, it was found that in worms, microtubules and dynein motors act through the nuclear envelope bridge formed by matefin/SUN-1 and ZYG-12, and that these components are at the core of licensing synapsis only for properly aligned bivalents [29].

Progression of meiosis is tightly regulated in *C. elegans*, and involves the checkpoint protein kinase CHK-2 at the meiotic entry. CHK-2 is responsible for the polarization of the chromatin, which is characteristic of the transition zone (TZ) [31], and is involved in induction of DSBs and proper SC polymerization, as well as the phosphorylation of SUN-1 [28]. PROM-1, an F-box–containing protein, controls progression of early meiosis; its depletion leads to an extended meiotic entry zone followed by nonhomologous synapsis [32]. The newly identified meiotic regulator HIM-19 is involved in chromatin polarization, formation of DSBs, and elongation of the SC. It encodes a protein with an RNA helicase domain, as determined by metastructure analysis [33]. In *cra-1* mutants, central-region components of the SC first fail to localize extensively to chromosomes; later, they instead polymerize along chromosomal axes, leading to unconnected bivalents. The tetratricopeptide repeat domain–bearing protein CRA-1 uncouples the polymerization of the central region components of the SC from the repair of DSBs [34].

In this study, we used live imaging microscopy to show that chromosome ends, highlighted as SUN-1 aggregates, were highly dynamic and contributed to chromosome movement in the leptotene/zygotene stages of *C. elegans* prophase I; they came together, coalesced and dispersed. Disruption of the SUN/KASH interaction in *mtf-1/sun-1(jf18)* mutants resulted in an absence of motion of the SUN-1 aggregates. We also analyzed the SUN-1-GFP aggregates in different mutant backgrounds affecting phosphorylation of SUN-1 and structural components of the lateral and central region of the SC, the DSB inducing enzyme SPO-11 and a group of genes that play a regulatory role during prophase I in *C. elegans*. Abrogation of synapsis led to a deceleration of SUN-1 aggregate movement. Aggregate behavior was influenced by recombination. Quantification of SUN-1 aggregates in meiotic regulators suggested that mere chromosome collisions driven by movement of nuclear envelope–attached chromosomes were insufficient for successful homologous pairing.

## Results

### Erratic movement of SUN-1 aggregates at one pole of the nucleus

*C. elegans* gonads recapitulate the progression of nuclei through meiotic prophase I in a spatial manner [35]. The mitotic zone is followed by the TZ, with its characteristically polarized chromatin (corresponding to leptotene/zygotene). In the next stage (pachytene), chromatin is redistributed and forms parallel tracks of DNA. Later, chromosomes condense (diplotene) and connected bivalents become apparent at diakinesis. SUN-1 forms foci and patches (the equivalent of the chromosomal attachment plaque seen in vertebrates) in the TZ [28]–[30].

**Figure 1:**
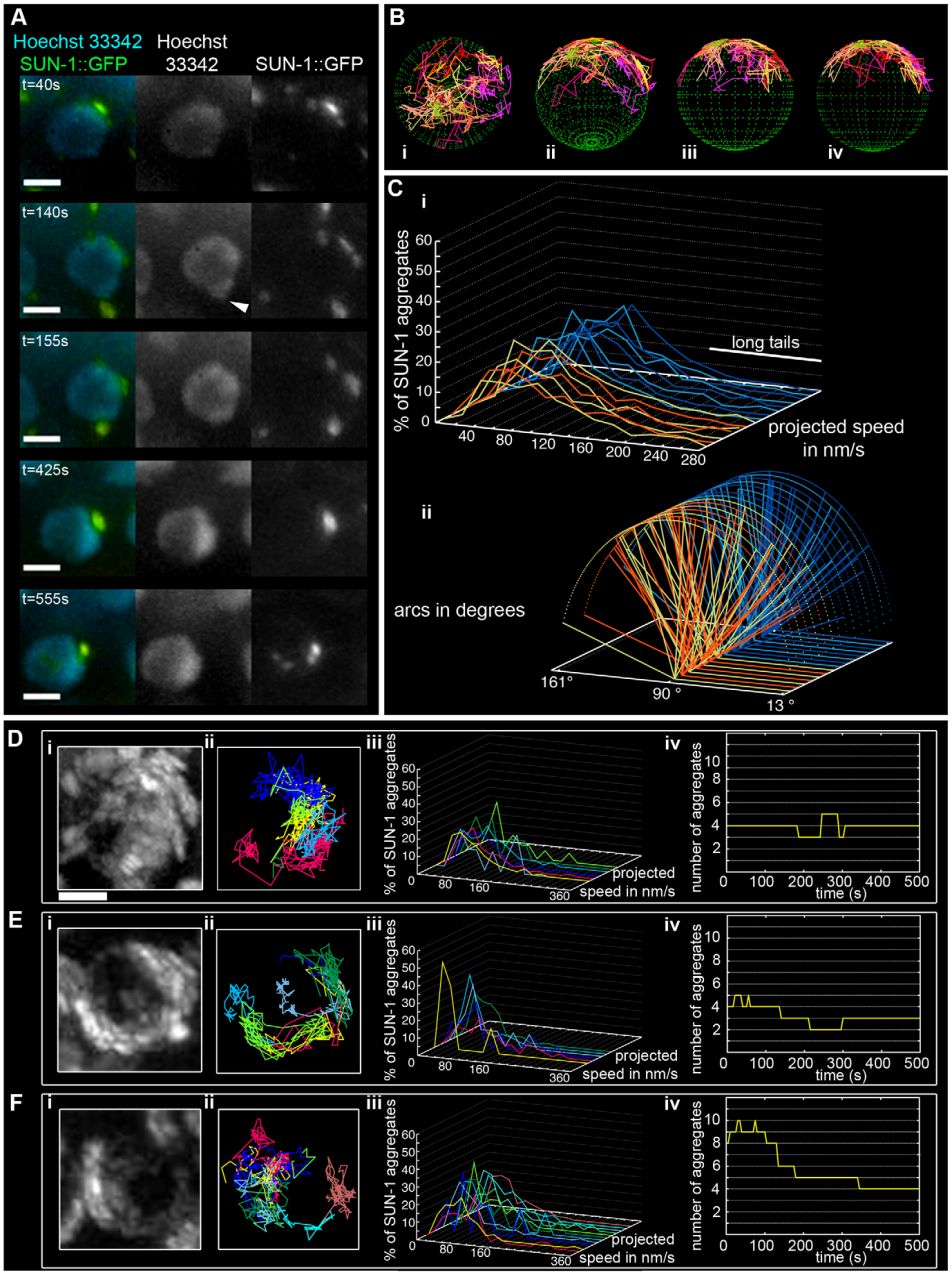
Dynamics of SUN-1 aggregates. (A) Frames from a movie showing the movement of SUN-1::GFP (green); chromatin stained with Hoechst 33342 (blue). White arrowhead highlights protrusion of chromatin. (B) Three-dimensional reconstruction of SUN-1::GFP displacement track dynamics. View from top (i), 30° z-axis rotation and 60° x-axis rotation (ii), and view from right side (iii) and left side (iv). (C) (i) Each line represents the distribution of the projected speed of all SUN-1 aggregates inside a nucleus from individual movies (yellow and orange: tracks from first movie, seven nuclei shown; light and dark blue: tracks from second movie, seven nuclei shown). (ii) Arcs show distance traveled for each SUN-1 track inside a nucleus. Yellow and orange: tracks from first movie, seven nuclei shown; light and dark blue: tracks from second movie, seven nuclei shown). Nuclei in the distal (D), central (E), and proximal (F) TZ, with projection of the cumulative movement of SUN-1::GFP (i), displacement tracks with different colors for each track (ii), distribution of speed for each track, using the same color code as for displacement tracks (iii), and number of SUN-1 aggregates as a function of time (iv). See Table 1 for number of nuclei analyzed. Scale bar: 2 µm.

We followed the movement of a functional SUN-1::GFP transgene integrated into the corresponding deletion background *sun-1(ok1282)* using in vivo imaging microscopy [28]. By additionally applying Hoechst 33342, we recorded both the motion of chromatin and the movement of SUN-1::GFP (Figure 1A, Video S1). SUN-1::GFP formed both large and small aggregates that moved in an erratic manner on one half of the nuclear periphery. Small and large aggregates met, fused and dispersed during the leptotene/zygotene stage of prophase I. Small aggregates, termed foci, likely define single chromosome end attachments, whereas large aggregates, termed patches, likely define multiple chromosome ends that are locally enriched. Indeed, only a single patch was visible at *t* = 425 s (Figure 1A), whereas at an earlier timepoint (*t* = 140 s), one patch and five foci were observed. Later (*t* = 555 s), we observed three foci and one patch. The coalescence of foci and patches were often very transient: in less than 130 s, we observed three foci driven out from the patch. Once two foci or one focus and a patch fused, they dispersed within a short time.

SUN-1::GFP aggregates, via cytoplasmic forces, also triggered protrusion of the chromatin, deforming the nuclear membranes. At *t* = 140 s, the chromatin was pulled outward by the SUN-1::GFP patch, which moved at the periphery of the nucleus (Figure 1A, white arrow; *t* = 140 s).

### SUN-1 aggregates are highly dynamic

#### General features of SUN-1 aggregates

Because of unreliable Hoechst staining, as reported by [7], only SUN-1::GFP dynamics were recorded during this study. To study the dynamics of SUN-1 aggregates, we manually followed their movement and characterized features such as the number of aggregates, distribution of the projected speed, the distance traveled, number of fusion/splitting events, and coalescence time. No distinction was made between patches and foci when tracking their movement; therefore, both SUN-1 patches and foci are referred to as aggregates.

Throughout the entire TZ, there were a minimum of 2.4±1.1 (standard deviation [SD], *n* = 27 nuclei) aggregates and a maximum of 6.1±2.8 (SD, *n* = 27 nuclei) aggregates. During the recorded time, on average, 3.9±1.4 (SD, *n* = 27 nuclei) aggregates (Table 1) per frame moved in an erratic manner over the surface of the nuclear membrane of single wild-type nuclei throughout the TZ (Video S2). The movement of SUN-1 aggregates was restricted to one hemisphere of the nucleus (Figure 1Di, 1Ei, and 1Fi; Video S1). The resulting displacement tracks of SUN-1 aggregates overlapped. In most cases, the projected movement of SUN-1 aggregates on the surface of the nucleus resembled the polarized conformation of the chromatin characteristic of leptotene/zygotene (Figure 1Dii, 1Eii, and 1Fii). In rare cases, the movement of the attachment plaques was observed from the top of the nuclei (Figure 1Bi). We reconstructed the 3D movement by considering that aggregates traveled only on one hemisphere of a spherical nucleus. (Figure 1Bi–iv). This reconstructed movement displayed undirected movement of chromosome ends. In addition, SUN-1::GFP aggregates moved outward toward the boundary of the nuclei (Figure 1Bii), (Figure 1A, *t* = 140 s), leading to bulging of the nuclear membrane. Aggregates usually moved randomly over short distances, but occasionally they traveled longer distances (Figure 1B).

**Table 1:** Numbers of aggregates in the region proximal to the mitotic zone for the different genotypes.

		number of aggregates			
		average	minimum	maximum	n
SUN-1::GFP	entire TZ	3.9±1.4	2.4±1.1	6.1±2.8	27
	distal part of TZ	3.5±1.1	2.1±0.7	5.7±2.0	10
	central part of TZ	4.6±1.1	2.3±1.6	7.5±2.5	4
	proximal part of TZ	3.9±1.6	2.1±0.9	5.4±3.0	9
SUN-1(G311V)::GFP		7.8±2.3	7.8±2.3	7.8±2.3	8
*him-3(gk149)*; SUN-1::GFP		1.1±0.3	1.1±0.3	1.1±0.3	82
*htp-1(gk174)*; SUN-1::GFP		3.1±08	2.6±1.0	3.9±1.0	14
*syp-2(ok307)*; SUN-1::GFP	distal part	2.8±1.0	1.7±1	3.5±1.1	30
	proximal part	3.3±0.9	2.0±1.0	4.2±1.2	45
*syp-3(me42)*; SUN-1::GFP	entire TZ	3.0±0.9	1.7±0.6	4.3±1.4	14
*htp-1(gk149)*; *syp-1(RNAi)*; SUN-1::GFP	distal part of TZ	3.5±0.6	2.4±0.8	4.5±0.9	23
	proximal part of TZ	2.8±0.6	2.2±0.6	3.3±0.8	24
*spo-11(me44)*; SUN-1::GFP	entire TZ	3.0±0.6	1.9±0.6	4.1±0.9	18
*spo-11(me44)*; SUN-1::GFP irradiated	entire TZ	3.6±0.6	2.3±0.6	4.7±0.9	23
*prom-1(ok1140)*; SUN-1::GFP	dispersed nuclei	3.4±0.9	2.7±0.9	3.8±1.1	37
*him-19(jf6)*; SUN-1::GFP	dispersed nuclei	2.8±0.8	1.9±0.6	4.0±1.3	8
*him-19(jf6)*; SUN-1::GFP irradiated	entire TZ	3.9±1.5	3.2±1.7	4.7±1.5	34
SUN-1::GFP irradiated	entire TZ	3.7±0.9	2.8±1.0	4.6±1.1	24
*cra-1(tm2144)*; SUN-1::GFP	entire TZ	3.7±0.7	2.3±0.8	5.4±1.0	22

The variations indicated correspond to the standard deviation. It should be noted that the number of nuclei analyzed for SUN-1::GFP in the distal, central, and proximal parts of the TZ do not add up to the number of nuclei for the entire TZ, because nuclei that where localized in the middle of these zones (distal and central or central and proximal) were not assigned to any specific part.

*n*, number of nuclei.

We also defined background motion in the filmed germ cells. To assess background motion, we measured the movement of SUN-1::GFP aggregates in animals killed with sodium azide. The values for the projected speed defined the background projected speed between 0 and 40 nm/s (Figure S1A).

The projected speed of SUN-1::GFP aggregates did not display a normal distribution, but rather a “Maxwellian-shaped” distribution (Figure 1Ci; see Figure S1B for an explanation of the term). Overall, more than half of the SUN-1 aggregates inside a nucleus (66%) moved within a range of 40–160 nm/s; approximately 10% of the aggregates showed a higher projected speed of up to 260 nm/s (long tails), and 24% were in the range of the background projected speed (*n* = 27 nuclei, taken from two movies, illustrated by the orange and blue colors in the figures).

To analyze the distance traveled, we extrapolated that the aggregates were moving on the periphery of a circle (projected nucleus), and measured the angle formed by the most extreme points of the tracks (Figure S2). Arcs do not express the absolute distance traveled; instead, they measure the area covered on the circle (a measurement of how vigorously aggregates move). We followed the arc for each track inside a nucleus, and also recorded the overall minimum and maximum values for the observed arcs (Figure 1Cii). Note that 180° is placed on the left of the x-axis, because angles were computed in a trigonometric circle. When the aggregate moved more than 180°/half plane, the radius of the circle increased, and the line representing this “arc” is dashed. Arc values for SUN-1 aggregates were not centered, but were evenly distributed between 13° and 163° (Figure 1Cii).

**Figure 2:**
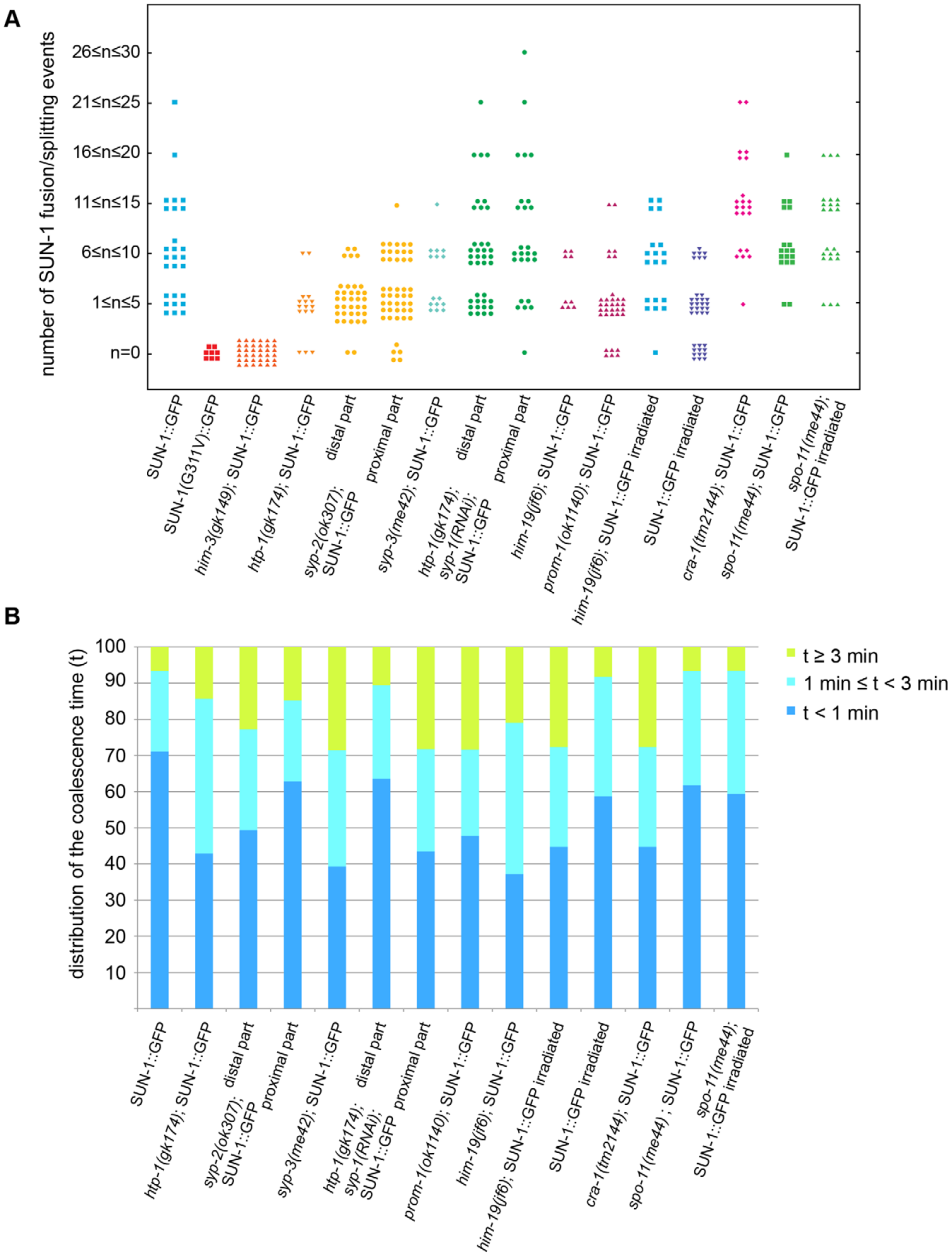
Dynamics of fusion/splitting events of SUN-1 aggregates for all genotypes studied (15 min recording). (A) Number of SUN-1 fusion/splitting events grouped into classes. (B) Quantification of the coalescence time (*t*) grouped into classes (*t*<1 min, 1 min≤*t*<3 min, and *t*≥3 min).

Because SUN-1 aggregates appeared to transiently coalesce (Figure 1A), we counted the number of fusion or splitting events of the aggregates during the first 15 min of the recordings (Figure 2A). The two most representative classes for the number of fusion/splitting events were 1–5 and 6–10 fusion/splitting events during the 15 min measuring period. A representative number of nuclei displayed between 11–15 fusion/splitting events, with an observed maximum of 25 exchanges. To assess how often exchanges take place, we looked at the time period between two fusion/splitting events. The “coalescence time” between aggregates was <1 min before splitting/fusing in 71% of cases, 1–3 min in 22% of cases, and >3 min in 7% of cases (Figure 2B).

Wild-type SUN-1 aggregates were particularly dynamic: they were movement-competent (traveled a distance of up to 160°) and able to reach high projected speeds (>160 nm/s). They fused and dispersed with high frequency (up to 15 fusion/splitting events during a 15 min interval of filming) during the leptotene/zygotene stage.

#### SUN-1 aggregate dynamics are independent of the position of the nucleus inside the TZ

To analyze whether SUN-1 aggregate behavior changes as the homology search progresses, we compared the aggregates in different parts of the TZ: distal (first row of cells of the TZ), proximal (last row of cells of the TZ), and central (row of cells clearly in the middle of the TZ). The appearance of tracks of SUN-1 aggregates was similar in each case. First, they exhibited the crescent form characteristic of the leptotene/zygotene stage (Figure 1Dii, 1Eii, and 1Fii). Second, no distinctions among the nuclei (from different positions in the TZ) could be made when looking at the distribution of the projected speeds. Aggregates in the three nuclei shown displayed the two classes of projected speed distribution: with or without long tails (Figure 1Diii, 1Eiii, 1Fiii). Aggregate numbers increased and decreased during the recorded time, independent of their position inside the TZ (Figure 1Div, 1Eiv, and 1Fiv). The distance traveled did not show a distinct pattern corresponding to the position in the TZ (Figure S3). No differences were found in the number of aggregates exchanged or the periodicity of these exchanges (data not shown). Therefore, we concluded that SUN-1 aggregates showed identical behavior, irrespective of their position in the TZ and the progress of homologous chromosome pairing.

### Leptotene/zygotene characteristic chromatin polarization requires connection of the chromosome ends to the cytoskeleton via a functional SUN/KASH bridge

**Figure 3:**
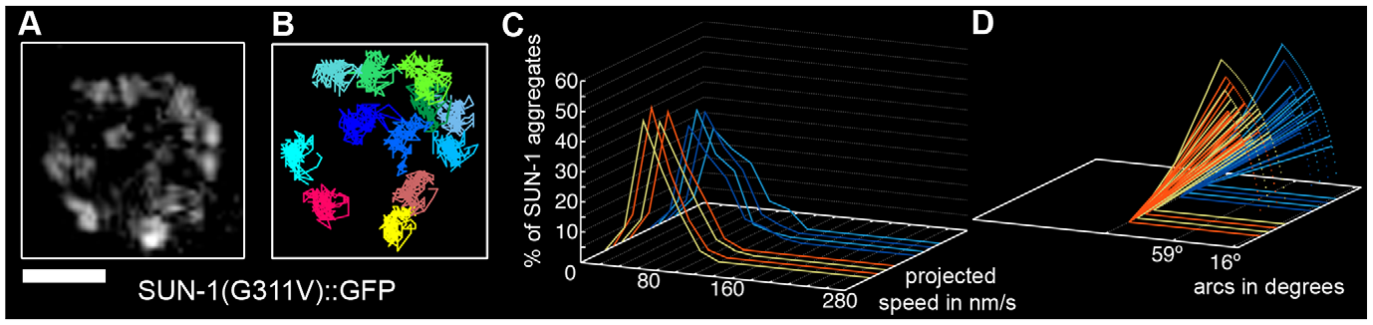
Disruption of the SUN/KASH bridge abrogates SUN-1 aggregate movement. Restrained movement in SUN-1(G311V)::GFP (A), displacement tracks (B), distribution of the projected speed of all SUN-1 aggregates inside a nucleus (C), and arcs representing the traveled distance for each track inside a nucleus (D). Blue lines represent values from the first movie, orange lines from the second. Eight out of the eight nuclei analyzed are shown. See Table 1 for number of nuclei analyzed. Scale bar: 2 µm.

A point mutation in the SUN domain of SUN-1 results in the absence of a defined TZ, and disturbs the interaction of SUN-1 with ZYG-12 [30]. In the cytoplasm, ZYG-12 interacts with the cytoskeleton via a dynein motor [29], [36]. We performed a time-lapse analysis of the mutated SUN-1(G311V)::GFP transgenic line in the *sun-1(ok1282)* deletion background and found that, on average, 7.8±2.3 (SD) aggregates inside a nucleus exhibited restrained movement (Table 1, Figure 3A and 3B, and Video S3). The distribution of the projected speed of these aggregates was a sharp bell, with 95% of the aggregates moving within a range of 10–100 nm/s (Figure 3C); the projected speed was significantly reduced compared to wild-type aggregates (Mann-Whitney test, *p*<0.001). The displacement tracks indicated weak local oscillatory movement (Figure 3B), and the distances traveled were significantly reduced. The arcs varied between 27° and 59° (Figure 3D). No exchange of SUN-1 aggregates was seen (Figure 2A). A functional SUN/KASH bridge mediating the connection to the cytoskeleton was, thus, necessary for the movement of SUN-1 aggregates and correlated with the absence of polarized chromatin.

### Loading of SC components is required for the formation of functional SUN-1 aggregates

The SC is a “zipper-like” structure that stabilizes the close pairing of parental chromosomes. This complex is composed of lateral elements on chromosome axes bridged by central region components [18]. We explored the behavior of SUN-1 aggregates in the SC mutants *him-3(gk149)*, *htp-1(gk174)*, *syp-2(ok307)*, and *syp-3(me42)*. HIM-3 is a major constituent of the lateral element, and HTP-1, in addition to being part of the lateral element, licenses synapsis [20], [21]. The central region components of the SC, SYP-1, SYP-2, SYP-3 and SYP-4 zip up paired homologs [37], [38], [19]. An allele of the central region component SYP-3 revealed that SYP-3 plays a role in meiotic repair pathway decisions, in addition to its role in SC formation [39].

**Figure 4:**
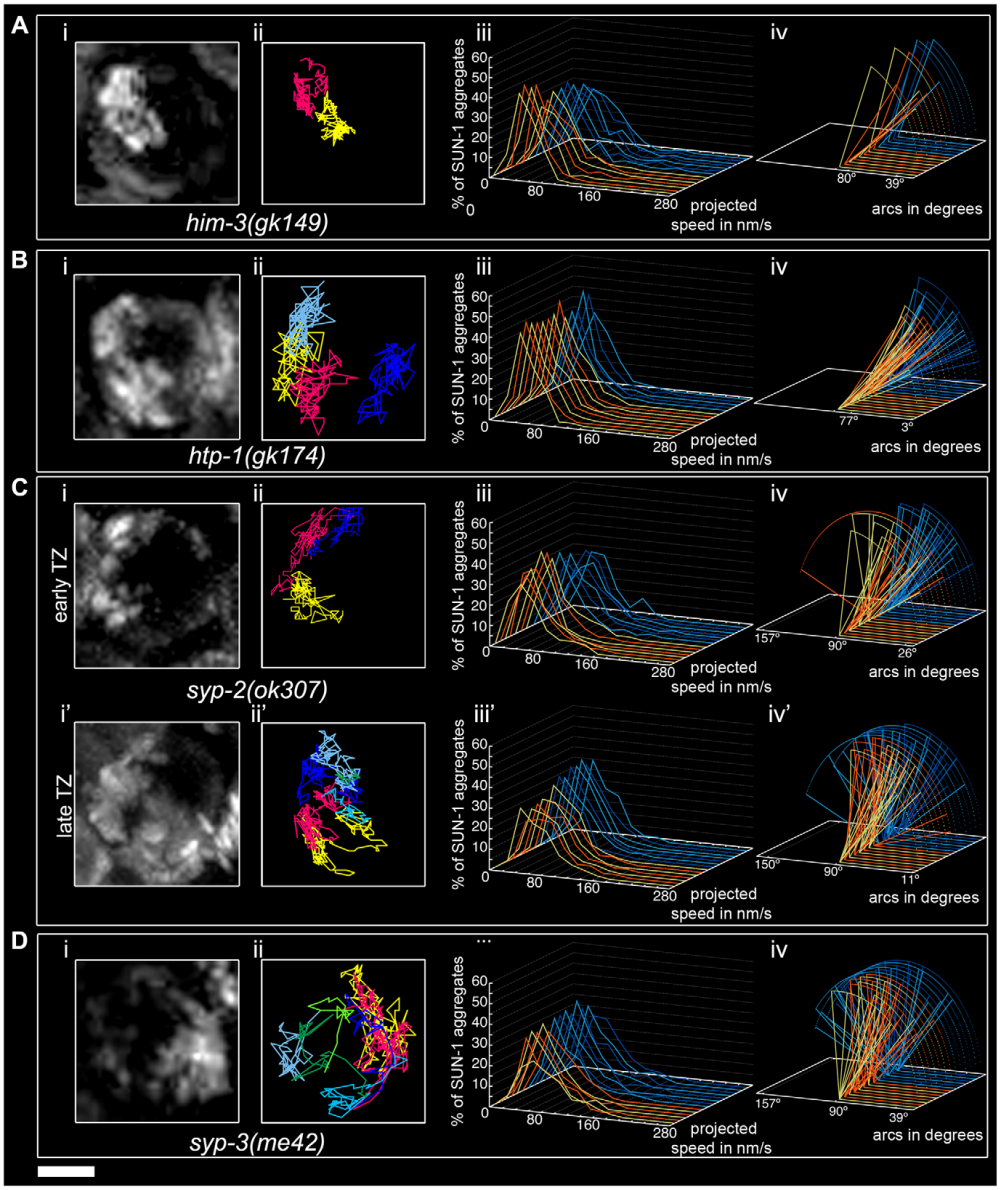
Impact of SC components on the dynamics of SUN-1 aggregates. *him-3(gk149)* (A), *htp-1(gk174)* (B), *syp-2(ok307)* (C), and *syp-3(me42)* (D), with projection of cumulative movement (i), displacement tracks (ii), and distribution of the projected speed (iii). Arcs represent traveled distance (iv). Blue lines represent values from the first movie, orange lines from the second. (C) *syp-2(ok307)* (i), (ii), (iii), and (iv) from the distal part of the extended TZ and i', ii', iii', and iv' from the proximal part. See Table 1 for number of nuclei analyzed. Scale bar: 2 µm.

The *him-3(gk149)* null allele displays loss of the polarized conformation of chromatin in the TZ of the gonad and lacks presynaptic alignment [40]. Depleting HIM-3 resulted in 1.1±0.3 (SD, *n* = 53) SUN-1 aggregates moving inside a nucleus (Table 1), and, on rare occasions, up to two aggregates (Figure 4Ai and 4Aii) unable to fuse (Figure 2A, Video S4). The projected speed distribution of the aggregates was “Gaussian-shaped,” with 95% of the aggregates moving within a range of 10–100 nm/s (Figure 4Aiii); this was significantly reduced compared to wild type (Mann-Whitney test, *p*<0.001). Depletion of HIM-3 also reduced the distance traveled by the SUN-1 aggregates: arc values only varied between 23° and 91° (Figure 4Aiv). HIM-8 is one of the four PC proteins, and binds specifically to the X chromosome [25]. In *him-3(gk149)*, HIM-8 always colocalizes with SUN-1::GFP, as shown by immunostaining [28]. Thus, the single SUN-1 aggregate moving with a reduced projected speed corresponds to the X chromosome and HIM-3 is, therefore, required for the formation of autosomal chromosome attachment plaques ([28] and this study). To test whether the formation of SUN-1 aggregates was impaired in *him-3(gk149)* due to mislocalization of the PC proteins, we stained *him-3(gk149)* with the PC protein ZIM-3 (marker for chromosomes I and IV). In *him-3(gk149)* mutant worms, the PC protein ZIM-3 did not colocalize with SUN-1 aggregates, although chromatin-associated signals were observed (Figure S4, Text S2). Therefore, we hypothesize that defective lateral elements of the SC impeded functional attachment plaque formation.

*htp-1* mutants precociously synapse with the wrong partner despite proper loading of HIM-3 [20], [21]. In *htp-1(gk174)*, the average number of aggregates was reduced (Mann-Whitney test, *p*<0.05). The maximum number of aggregates was comparable to the average number of SUN-1 aggregates in wild-type worms (Table 1). The displacement tracks of SUN-1::GFP in this background recapitulated the polarization of the chromatin characteristic of the TZ (Figure 4Bi and 4Bii; Video S5). The projected speed distribution within a nucleus was comparable to that in *him-3(gk149)*. For most SUN-1 aggregates (>95%), the projected speed varied between 10 and 100 nm/s (Figure 4Biii). The distance traveled was significantly reduced compared to *him-3(gk149)* (Mann-Whitney test, *p*<0.001): arc values ranged from 20° to 62° (Figure 4Biv). The precocious synapsis in *htp-1(gk174)* mutant worms likely inhibits the mobility of SUN-1 aggregates (see below). This interpretation was reinforced by a significant decrease in the number of fusion/splitting events (Figure 2A and Table S1) and a significant increase in the time without exchange (Figure 2B and Table S2). Indeed, SUN-1 aggregates coalesced for longer than 1 min in 60% of cases. Although ZIM-3 loading was defective in *him-3(gk149)*, it was unaffected in *htp-1(gk174)*, with 2–4 ZIM-3 signals overlapping with SUN-1 aggregates (Figure S4). We conclude that *htp-1(gk174)* mutant worms readily formed single chromosomal attachment plaques.

The SC central region component mutant *syp-2(ok307)* has an extended TZ, characteristic of SC mutants [38]. We compared the dynamics of SUN-1 aggregates in distal (first half of the TZ, Video S6) and proximal (second half of the TZ, Video S7) positions in the prolonged TZ. In the distal part, on average, 2.8±1 (SD, *n* = 30) SUN-1 aggregates showed reduced movement (Figure 4Ci and 4Cii); in addition, the maximum number of SUN-1 aggregates was reduced (3.5±1.1; SD, *n* = 30) compared to wild type (Table 1; Mann-Whitney test, *p*<0.05). Although the projected speed distribution of 95% of SUN-1 aggregates was 10–100 nm/s (Figure 4Ciii), comparable to *him-3(gk149)*, the distribution was more Maxwellian-shaped. The distance traveled by SUN-1::GFP aggregates was reduced (from 16° up to 90°, with the exception of one track that went up to 163°; Figure 4Civ) compared to wild type. Depletion of SYP-2 also reduced the number of SUN-1::GFP fusion/splitting events in the distal part of the TZ (Figure 2A and Table S1), with mostly 1–5 fusion/splitting events occurring within 15 min. The time of coalescence between SUN-1 aggregates was increased. Indeed, only 50% of SUN-1 aggregates coalesced for less than 1 min, while 22% of them coalesced for more than 3 min (Figure 2B and Table S2).

*syp-2(ok307)* SUN-1::GFP aggregates in the proximal part of the TZ traveled longer distances. The arcs were between 20° and 150° (Figure 4Civ'), which is in accordance with a broader projected speed distribution. The projected speed distribution for 95% of the aggregates ranged between 15 and 140 nm/s (Figure 4Ciii'), and adopted a more Maxwellian-shaped distribution than in the early TZ, with the distribution shifted towards the higher speed. In the proximal part of the TZ, the displacement tracks were more reminiscent of the crescent shape of the chromatin than in the distal part of the TZ (compare Figure 4Ci and 4Ci'). In the proximal part of the TZ, the maximum number of SUN-1 aggregates went up to 4.2±1.2 (SD, *n* = 45) (Table 1); nevertheless, their average number remained decreased compared to wild type (Mann-Whitney test, *p*<0.05). The number of SUN-1::GFP fusion/splitting events also differed from those seen in wild-type worms; there were less than ten exchanges within 15 min (Figure 2A and Table S1). The periodicity of the exchanges was similar to wild type (Figure 2B and Table S2). Disruption of synapsis resulted in a lack of long tails in the speed distribution and a decrease in the number of SUN-1 aggregates exchanged in both the distal and proximal parts of the TZ.

The *syp-3* allele *me42* is special because it has a shortened TZ and appears to use a different repair pathway for the repair of meiotic DSBs [39]. In contrast to SC-deficient mutants, in *me42*, the central region component SYP-1 was found to be polymerized on univalents. The displacement tracks of SUN-1::GFP in *syp-3(me42)* followed a half-moon shape (Figure 4Di and 4Dii; Video S8). The number of SUN-1 aggregates was significantly decreased compared to wild-type values (Table 1; Mann-Whitney test, *p*<0.001). The distribution of the projected speed was more Maxwellian-shaped and comparable to the distribution of SUN-1::GFP aggregates in the distal part of the *syp-2(ok307)* mutants (Figure 4Diii). The distance traveled was greater than in *syp-2(ok307)* mutant worms, and covered angles from 37° to 100° (Figure 4Div). The number of SUN-1::GFP fusion/splitting events was comparable to those in wild-type worms (Figure 2A and Table S1), but the frequency of the exchanges was significantly decreased (Figure 2B and Table S2).

**Figure 5:**
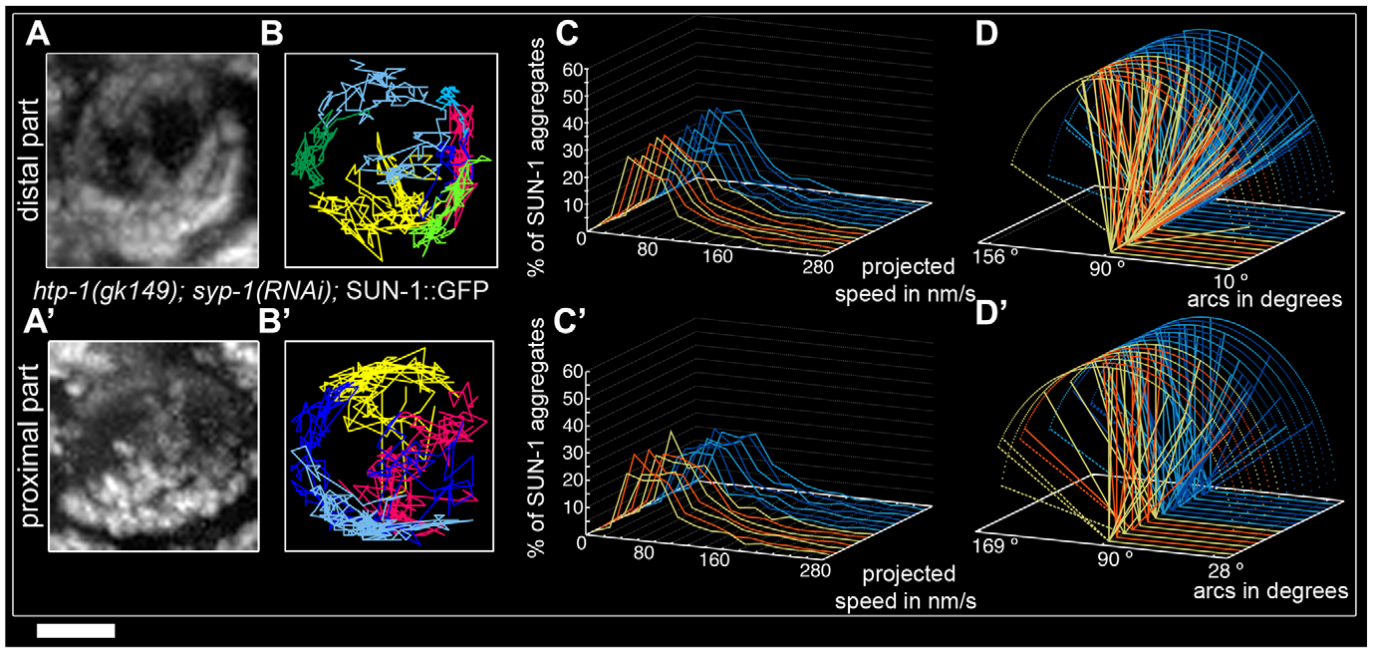
Restrained movement of SUN-1 aggregates in *htp-1(gk174)* is due to precocious synapsis. Projection of the cumulative movement of SUN-1::GFP in *htp-1(gk174)*; *syp-1(RNAi)* (A, A'), displacement tracks (B, B'), distribution of the projected speed (C, C'), and arcs (D, D'). Blue lines represent values from the first movie; orange lines values from the second. (A, B, C, D) from distal TZ, (A', B', C', D') from proximal zone where SUN-1 aggregates move. See Table 1 for number of nuclei analyzed. Scale bar: 2 µm.

To test the idea that restricted aggregate behavior in *htp-1(gk174)* was due to nonhomologous synapsis, we depleted SYP-1 in *htp-1(gk174)* mutants. Surprisingly, in *htp-1(gk174)*; *syp-1(RNAi)* SUN-1 aggregates were extended and not restricted to the first cell row where chromatin is strongly polarized (Figure S5). SUN-1 aggregates were still detectable in nuclei with more loosely clustered chromatin. We divided the zone with aggregates into distal (Video S9) and proximal (Video S10) for analysis. In the distal part of *htp-1(gk174)*; *syp-1(RNAi)* the average number of SUN-1 aggregates (3.5±0.6, SD, n = 23, Table 1) was close to wild type, but their maximum number was significantly reduced (Mann-Whitney test, *p*<0.05). Displacement tracks adopted a circular form, in contrast to the crescent shape seen in wild type (Figure 5A and 5B, 11 out of 23 nuclei), where movement pushes the nucleolus to one side of the nucleus. Here the tracks are found at the periphery, with chromatin likely rotating around the nucleolus. SYP-1 depletion in *htp-1(gk174)* significantly increased the projected speed of SUN-1 aggregates when compared to *htp-1(gk174)*, nevertheless the percentage of long tails (4%) was significantly below wild-type values (Figure 5C, Mann-Whitney test, *p*<0.05). The distance traveled was also significantly increased in *htp-1(gk174)*; *syp-1(RNAi)*, with arc values reaching 156° (compare Figure 4Biv and Figure 5D, Mann-Whitney test, *p*<0.05). Depletion of SYP-1 in *htp-1(gk174)* resulted in an increase of exchanged aggregates (Figure 2A, Table S1) and restored the periodicity of these exchanges to the wild type value (Figure 2B, Table S2).

In the proximal region of *htp-1(gk174)*; *syp-1(RNAi)* gonads the number of SUN-1 aggregates was significantly reduced compared to the distal part (Mann-Whitney test, *p*<0.05) with an average of 2.8±0.6 (SD, n = 24, Table 1). Of the 23 nuclei analyzed, 16 nuclei displayed circular displacement tracks (Figure 5A' and 5B'). The distribution of the projected speed was significantly increased compared to the distal part (6% long tails, Figure 5C', Mann-Whitney test, *p*<0.05), but still below wild-type values (Mann-Whitney test, *p*<0.05). SUN-1 aggregates traveled longer distances in the proximal part than in the distal (Figure 5D', Mann-Whitney test, *p*<0.05), but the distances traveled, the exchanges, and the frequencies were still below wild-type values (Mann-Whitney test, *p*<0.05). (Figure 2A and 2B, Tables S1 and S2).

Our analysis of the behavior of SUN-1 aggregates in mutants defective in SC formation confirmed that intact lateral elements of the SC were necessary for the formation of functional SUN-1 aggregates. We showed that SC components played a role in the exchange of SUN-1 aggregates. HTP-1 has an inhibitory influence on the exchange of aggregates as we observed reduced exchanges in *htp-1(gk174)* and *syp-2(ok307)* single mutants, whereas exchanges were increased to wild type values in *htp-1(gk174)*; *syp-1(RNAi)*). Strikingly, defects in SC polymerization reduced the projected speed of aggregates, whereas decreasing synapsis in the *htp-1* mutant increased the projected speed of SUN-1 aggregates. The persistent aggregates in the *syp-1 htp-1* double mutant formed circular displacement tracks concomitant with only loosely clustered chromatin.

### Modulation of SUN-1 aggregate properties by meiotic regulators CHK-2, HIM-19, PROM-1, and CRA-1

To date, known early meiotic regulators with a role in chromosome pairing in *C. elegans* include CHK-2, HIM-19, PROM-1, and CRA-1. Depletion of CHK-2 results in a lack of SUN-1 aggregates in *chk-2* [28]; therefore, no live imaging can be shown. HIM-19 is a newly identified meiotic regulator. In *him-19(jf6)* mutants, meiotic defects are aggravated with age. In 2-day-old (2-d-old) *him-19(jf6)* mutant worms, the TZ is not defined, DSB formation is likely defective, and elongation of the SC is restricted and nonhomologous [33]. PROM-1 is involved in the progression of meiosis I, and its depletion elicits nonhomologous synapsis. *prom-1(ok1140)* mutant worms lack a defined TZ and instead display dispersed nuclei with a polarized conformation after a prolonged meiotic entry zone [32]. CRA-1 regulates SC formation. Unlike *chk-2*, *prom-1*, and *him-19*, *cra-1* mutant worms display an extended TZ. In addition, *cra-1(tm2144)* mutant worms are defective in the formation of the SC central region [34].

**Figure 6:**
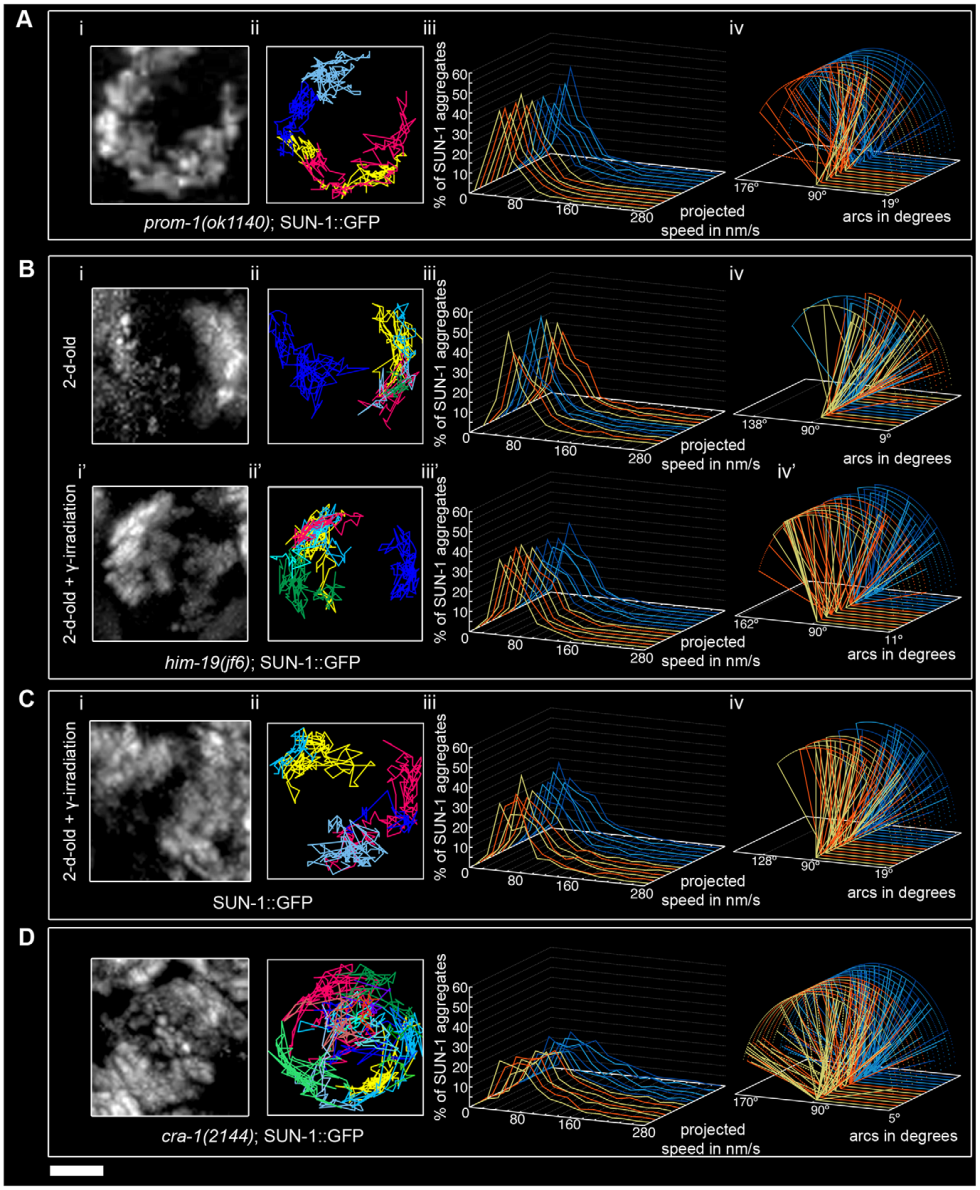
Influence of meiotic regulators on dynamics of SUN-1 aggregates. *prom-1(ok1140)* (A), *him-19(jf6)* (B), irradiated wild type (C), and *cra-1(tm2144)* (D), showing projection of the cumulative movement of SUN-1::GFP aggregates (i), displacement tracks (ii), distribution of the projected speed (iii), and arcs representing travelled distances (iv). Blue lines represent values from the first movie, orange lines from the second. See Table 1 for number of nuclei analyzed. Scale bar: 2 µm.

In dispersed nuclei with a TZ-like appearance in *prom-1(ok1140)* mutant worms, SUN-1::GFP aggregates were movement competent (Video S11), and their tracking reconstructed the crescent shape of the chromatin (Figure 6Ai and 6Aii). The number of SUN-1 aggregates in *prom-1(ok1140)* was reduced compared to wild type (Mann-Whitney test, *p*<0.05), despite a similar average number of aggregates (Table 1). In this background, SUN-1 aggregates displayed a Maxwellian-shaped projected speed distribution and lacked long tails (Figure 6Aiii; Mann-Whitney test, *p*<0.05 compared to wild type), although the distance traveled by SUN-1::GFP aggregates was unaffected (from 19° up to 143°; Figure 6Aiv; Mann-Whitney test, *p*>0.05). Depletion of PROM-1 also reduced the number of exchanged aggregates (Figure 2A). Indeed, in a significant number of nuclei, SUN-1 aggregates were unable to fuse or split, and the number of nuclei showing 6–10 fusion/splitting events was considerably reduced (Table S1). The time period without exchange was significantly increased in *prom-1(ok1140)* compared to wild type: 47% of SUN-1::GFP aggregates were found to coalesce for less than 1 min and 23% for more than 3 min (Figure 2B and Table S2).

In *prom-1(ok1140)*, it is likely that the delayed orchestration of the meiotic program led to a reduction in the speed of SUN-1 aggregates without affecting the distance traveled. In addition, the number and frequency of exchanges were reduced in *prom-1(ok1140)* mutants.

The formation of SUN-1 aggregates in 2-d-old *him-19(jf6)* mutants was restricted to the few dispersed nuclei with a polarized chromatin conformation, but could be augmented by γ-irradiation (Figure 6B and [28]). In aged *him-19(jf6)* worms, a few SUN-1::GFP aggregates were movement competent (Video S12), and their displacement tracks resembled a crescent shape (Figure 6Bi and 6Bii). Both the average number of SUN-1 aggregates (2.8±0.8; SD, *n* = 8)) and the maximum number of aggregates was decreased (4.0±1.3; SD, *n* = 8) (Table 1; Mann-Whitney test, *p*<0.05). In addition, the projected speed distribution of SUN-1::GFP aggregates was reduced compared to wild type (Mann-Whitney test, *p*<0.05): it was similar to the speed of SUN-1::GFP aggregates in *htp-1(gk174)*, with 95% of the aggregates moving from 10 to 130 nm/s (Figure 6Biii). The distance covered was also reduced, as demonstrated by arc values ranging from 9° up to 137° (Figure 6Biv; Mann-Whitney test, *p*<0.05). In *him-19(jf6)* mutants, the number of SUN-1 fusion/splitting events was wild type (Figure 2A and Table S1), whereas the time without exchange increased, with 38% of SUN-1 aggregates coalescing for less than 1 min and 21% for more than 3 min (Figure 2B and Table S2).

Two hours after γ-irradiation, the average number of SUN-1 aggregates reached wild-type levels in 2-d-old *him-19(jf6)* worms (Table 1); however, the number of SUN-1 aggregates was significantly reduced (Mann-Whitney test, *p*<0.05). The displacement tracks of SUN-1::GFP in 2-d-old irradiated *him-19(jf6)* worms recapitulated the polarized conformation of the chromatin (Figure 6Bi' and ii'; Video S13). The distribution of the projected speed of SUN-1::GFP aggregates was shifted towards the higher speed after irradiation and became more Maxwellian-shaped. Nevertheless, long tails were absent from the distribution (Figure 6Biii'). The projected speed of SUN-1 aggregates was reduced in aged irradiated *him-19(jf6)* compared to wild type (Mann-Whitney test, *p*<0.05). In contrast, irradiation-induced SUN-1::GFP aggregates in *him-19(jf6)* moved similar to those in wild-type worms in terms of the distance traveled (Figure 6Biv'; Mann-Whitney test, *p*>0.05), whereas the number of exchanges was significantly reduced. The class of 1–5 fusion/splitting events represented more than 50% of nuclei, and the exchange was abrogated for a representative number of nuclei (Figure 2A and Table S1). The frequency of these exchanges was reduced compared to wild type (Figure 2B and Table S2), and there was no significant increase compared to nonirradiated *him-19(jf6)* worms.

We conclude that γ-irradiation restored the formation of SUN-1 aggregates to wild-type levels with respect to their numbers and distances traveled, whereas the dynamics of SUN-1 aggregates remained impaired both in terms of the distribution of the projected speed and the number and frequency of exchanges.

To ensure that γ-irradiation had no side effects, we irradiated 2-d-old worms solely expressing SUN-1::GFP and performed the same analysis. The appearance of the displacement tracks of SUN-1::GFP aggregates was circular in 12 of the 24 nuclei analyzed whereas the other ones recapitulated the crescent shape of the chromatin (Figure 6Ci and 6Cii; Video S14). The distribution of the projected speed of SUN-1::GFP aggregates was shifted markedly towards lower values, with only 3% long tails (>160 nm/s) (Figure 6Ciii; Mann-Whitney test, *p*<0.05). Nonetheless, after γ-irradiation, SUN-1::GFP aggregates move faster in wild-type worms than in irradiated *him-19(jf6)* (Mann-Whitney test, *p*<0.05). In addition, the maximum number of SUN-1 aggregates was reduced (Table 1; Mann-Whitney test, *p*<0.05) compared to nonirradiated wild type. γ-irradiation also reduced the distance traveled by SUN-1 aggregates; arc values ranged between 19° and 128° (Figure 6Civ; Mann-Whitney test, *p*<0.05). γ-irradiation had no impact on the number of SUN-1::GFP fusion/splitting events (Figure 2A and Table S1). However, it significantly increased the time without exchanges (Figure 2B and Table S2). FISH analysis with a probe specific for chromosome V revealed that pairing was affected after γ-irradiation (Figure S6A and S6B). To ascertain whether the decrease in the speed distribution might be due to an SC defect, we stained for SYP-1 in nonirradiated and irradiated wild-type worms. No gross irregularities in SYP-1 polymerization were evident 2 h after γ-irradiation (Figure S6C, Text S2).

γ-irradiation clearly had an impact on the dynamics of SUN-1 aggregates in wild-type gonads. Nonetheless, the behavior of restored SUN-1 aggregates in irradiated *him-19(jf6)* mutants adopted a more wild-type-like behavior compared to the sparse aggregates in non-irradiated *him-19(jf6)* gonads.

In *cra-1(tm2144)* mutants, SUN-1 aggregates were movement competent (Video S15), and their displacement tracks adopted a rather circular form (19 out of 22), like in *htp-1(gk174); syp-1(RNAi)* (Figure 6Di and 6Dii). Likewise, in *cra-1(tm2144)* chromatin loosely clustered following a short stretch of nuclei with strong chromatin polarization (Figure S5). The number of SUN-1 aggregates was similar to that of wild type (Table 1; Mann-Whitney test, *p*>0.05). However, the distribution of the projected speeds of SUN-1 aggregates increased towards higher speeds (12% long tails) (Figure 6Diii; (Mann-Whitney test, *p*<0.05). This was also the case for the distance traveled, with arcs ranging from 15° up to 173° (Figure 6Div; Mann-Whitney test, *p*<0.05). Except for the class of 1–5 fusion/splitting events, which was significantly reduced compared to wild type, the number of SUN-1 fusion/splitting events was similar to that of wild type in the *cra-1(tm2144)* background (Figure 2A and Table S1). The frequency of SUN-1 aggregate exchanges was unaffected in *cra-1(tm2144)* (Figure 2B and Table S2).

In *cra-1(tm2144)*, the kinetics of SUN-1 aggregates were increased (speed and distance traveled) compared to wild type, whereas the number and frequency of fusion/splitting events was unaffected.

Impairment of meiotic regulators showed that DSBs could be involved in the formation of functional SUN-1 aggregates, and that γ-irradiation nonetheless had some impact on SUN-1 aggregate movement. Improper orchestration of the meiotic program disturbed SUN-1 aggregate dynamics.

### DSBs are required for wild-type SUN-1 aggregate behavior

In order to test whether recombination impacts SUN-1 aggregate dynamics, we followed SUN-1 movement in the *spo-11(me44)* mutant (Video S16).

**Figure 7:**
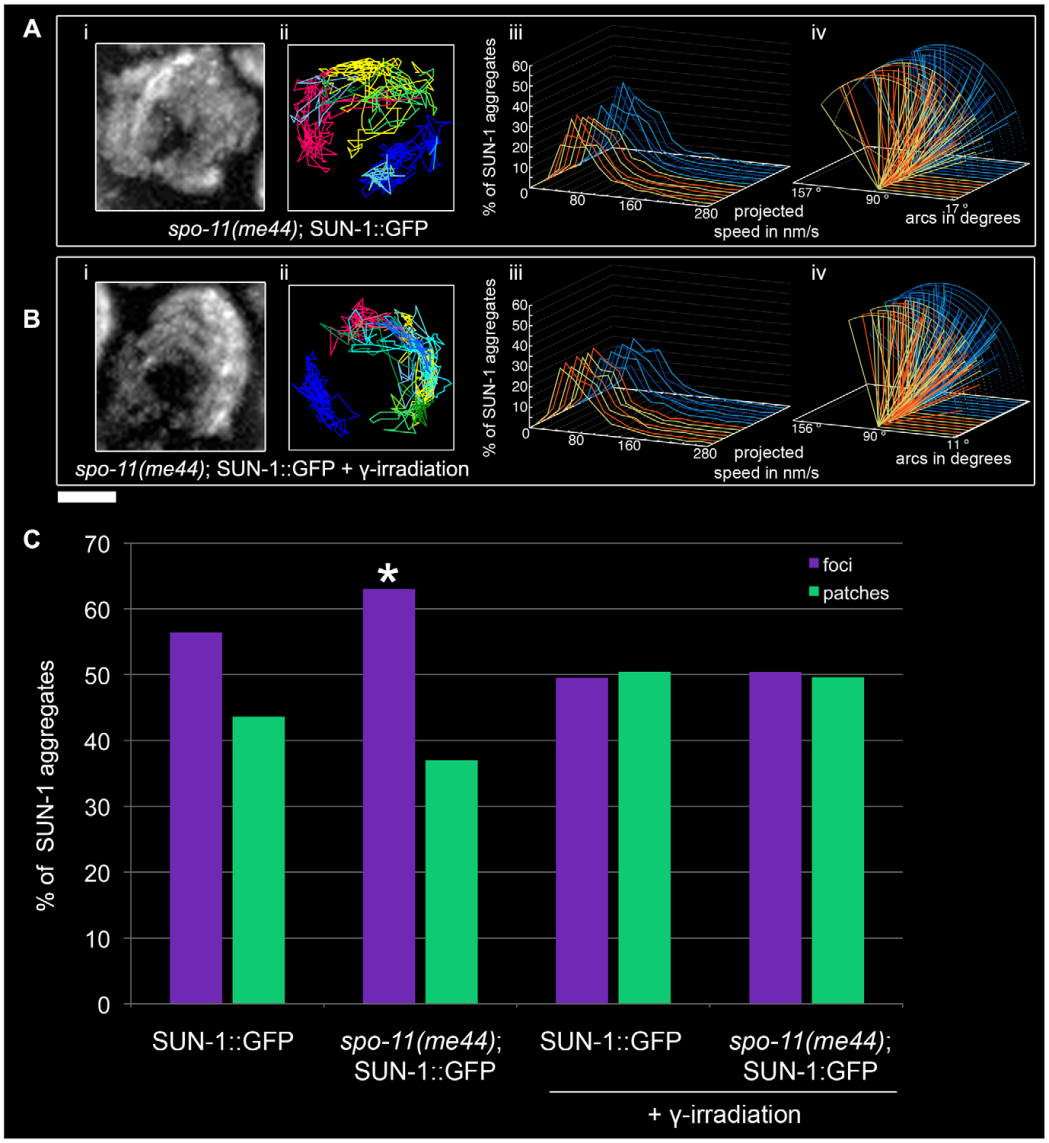
Effect of DSB formation on SUN-1 aggregate dynamics. *spo-11(me44)* (A), 2-d-old *spo-11(me44)* (B) 2 hours after irradiation with projection of cumulative movement (i), displacement tracks (ii), and distribution of the projected speed (iii). Arcs represent traveled distance (iv). Blue lines represent values from the first movie, orange lines from the second. See Table 1 for number of nuclei analyzed. (C) Distribution of SUN-1 foci and patches formed in wild type, *spo-11(me44)*, 2-d-old wild type 2 hours after irradiation and 2-d-old *spo-11(me44)* 2 hours after irradiation. >400 SUN-1 aggregates counted per genotype. Scale bar: 2 µm.

The average number of SUN-1 aggregates was significantly reduced (3.0±0.6, SD, n = 18, Table 1) compared to wild type. Surprisingly, the appearance of SUN-1 displacement tracks was circular in 9 out of the 18 nuclei analyzed (Figure 7Ai and 7Aii). The distribution of the projected speed of SUN-1 aggregates in *spo-11(me44)* showed only 5% long tails (Figure 7Aiii), and was reduced in terms of speed and distance traveled (Mann-Whitney test, *p*<0.05) (Figure 7Aiv). In *spo-11(me44),* the number and frequency of exchanges (Figure 2A and 2B, Tables S1 and S2) were wild type.

Next we tested whether introduction of artificial DSBs by γ-irradiation in *spo-11(me44)* (Video S17) could restore the properties of SUN-1 aggregates to wild-type values. The average number of SUN-1 aggregates (3.6±0.6, SD, n = 23, Table 1) in *spo-11(me44)* mutants increased to wild-type levels after irradiation. The displacement tracks were circular in 11 of the 23 nuclei analyzed (Figure 7Bi and 7Bii). The distribution of the projected speed of SUN-1 aggregates displayed 3% of long tails, like wild type 2 hours after irradiation, but SUN-1 aggregates tended to move faster (Mann-Whitney test, *p*<0.05). γ-irradiation had no impact on the distance traveled (Figure 7Biv, Mann-Whitney test, *p*>0.05). The frequency of exchanges was significantly reduced when compared to wild type (Figure 2B, Table S2), but no effect could be detected in the number of SUN-1 aggregates exchanged (Figure 2A, Table S1). The properties of SUN-1 aggregates in *spo-11(me44)* after irradiation were close to wild-type values after irradiation.

Intrigued by the fact that less SUN-1 aggregates can be found in *spo-11(me44)*, we compared the ratio of foci and patches in the gonad of both irradiated and non-irradiated wild type and *spo-11(me44)* worms. We found a significant increase in the number of foci in *spo-11(me44)* compared to wild type (Figure 7C, Fisher's exact test, p<0.05). The ratio of foci to patches was significantly reduced after irradiation (Figure 7C, Fisher's exact test, p<0.05) with an increase in the fraction of patches for wild type and *spo-11(me44)*.

All together, these results confirm that formation of DSBs is necessary for the generation of wild-type numbers of SUN-1 aggregates and patches. The appearance of circular tracks in *spo-11(me44)* confirms loose chromatin clustering in *spo-11(me44)* (not shown).

## Discussion

During early meiotic prophase, SUN-1 forms movement-competent aggregates that can be categorized into two classes: foci and patches. Foci most likely represent single chromosome end attachments, whereas patches harbor multiple chromosome ends in a local cluster, reminiscent of the chromosomal bouquet [28]. These aggregates fuse and disperse, with an average number of four aggregates during leptotene/zygotene, and cover distances on the order of half a circle after projecting movement into two dimensions. The movement of SUN-1 aggregates anchored in the inner nuclear membrane is able to induce outward directed protrusion of the chromatin and, thus, of the nuclear envelope, as in budding yeast [7] and maize [9].

### Formation of functional SUN-1 aggregates

Wild-type numbers of SUN-1 aggregates require HIM-3, a component of the lateral elements of the SC. The sole movement-competent aggregate in the *him-3(gk149)* mutant colocalizes with the X chromosome [28]. The “autosomal SUN-1 aggregates” are, therefore, missing. Lateral elements of the SC are possibly required for their formation; alternatively, they may affect their stability. The fact that formation of attachment plaques at the nuclear periphery is independent of the lateral element of the SC in rat testis [41] provides support for the latter explanation. The reduced mobility of the SUN-1 aggregate in *him-3(gk149)* could be explained by the ability of the lateral element of the SC to rigidify the chromosomes, thereby supporting resolution of chromatin entanglements that otherwise might slow down movement.

Formation of functional SUN-1 aggregates also requires a functional SUN-domain, enabling the movement of SUN-1 aggregates. We showed that disruption of the SUN/KASH bridge abrogated the movement of SUN-1 aggregates, leading to nonhomologous synapsis in SUN-1(G311V)::GFP. The movement of SUN-1 aggregates, thus, exerted an inhibitory action on synapsis with the wrong partners. In addition, SUN/KASH-mediated movement exerted a positive effect on synapsis by mixing chromosomes in the nucleus, thereby positively reinforcing homologous synapsis. The movement of SUN-1 aggregates also elicited the polarized appearance of the chromatin, which required more than one aggregate to be moving, as exemplified in *him-3(gk149)*, where chromatin did not cluster.

### Do SUN-1 patches solely reflect chromosome end shuffling as the driving force for pairing?

Chromosome ends move along the inner surface of the nuclear envelope and come together in areas where patches of SUN-1 are seen. At the same time, SC polymerization is present, implying that once parental partner chromosomes have met, they engage in synapsis [37]. At this point, successful synapsis did not lead to dissolution of the aggregate. There was no difference in terms of the number of SUN-1 aggregates during the progression of leptotene/zygotene (as assessed by dividing this stage into three substages); instead, the average number of SUN-1 aggregates fluctuated at around four.

**Figure 8:**
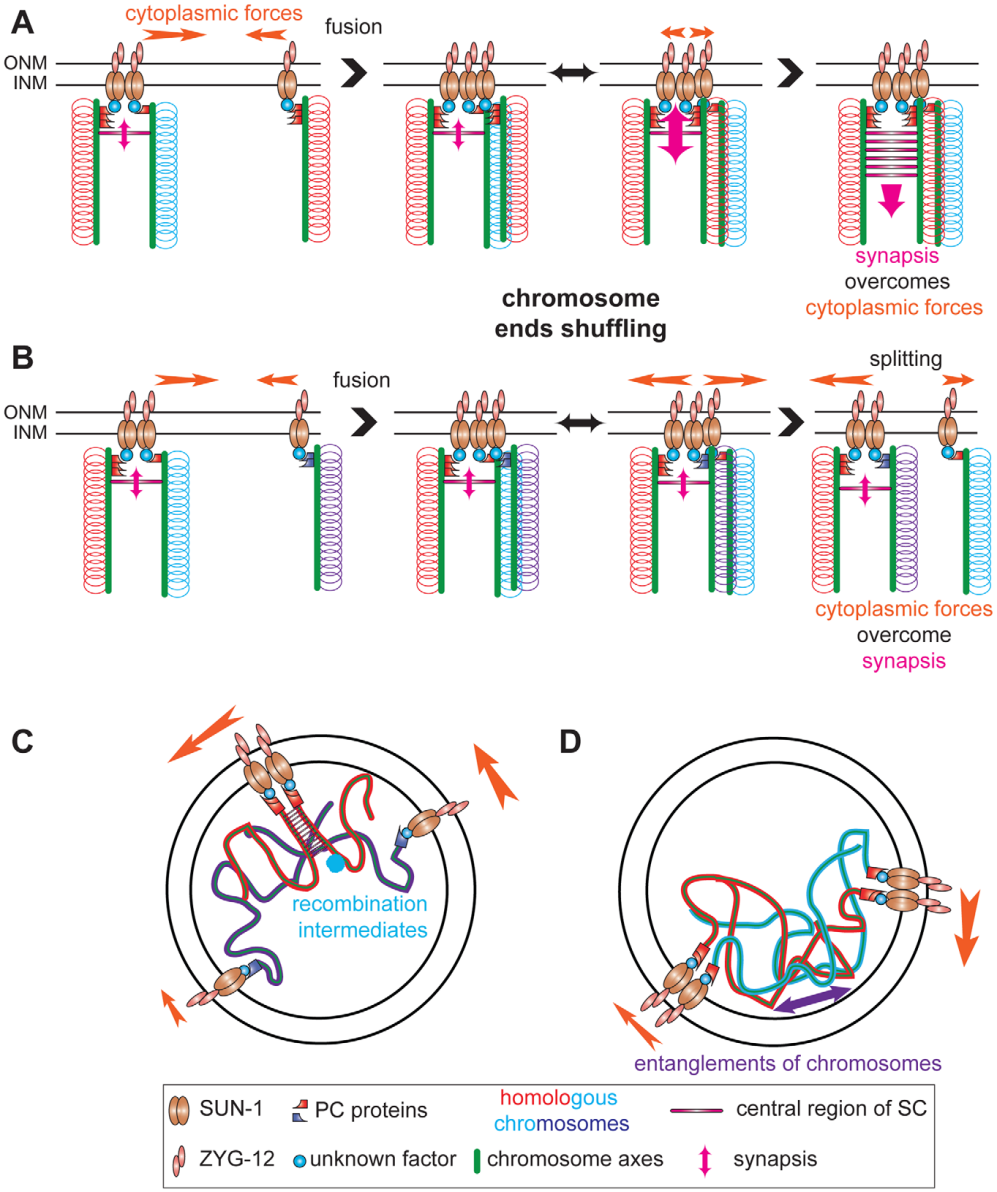
Establishment of synapsis and formation of high speed via shuffling of chromosome ends through SUN-1 patches. Chromosome axes (green lines) support binding of PC proteins (red or violet shapes) that connect chromosome ends (red, blue, and violet loops) to SUN-1 (brown ellipses), directly or indirectly, (unknown factor, blue circle). ZYG-12 (pink ellipses) and SUN-1 bridge chromosomes to cytoplasmic forces (orange arrow) to move chromosome ends. (A) SUN-1 patch containing two nonhomologous chromosome ends (red and blue loops) fuses with SUN-1 focus carrying a single chromosome end, a homolog (red loops). After fusion, chromosome ends will be shuffled inside the newly formed SUN-1 patch. When the homologous chromosome is found, synapsis overcomes the cytoplasmic forces and synapsis can be established. (B) The same scenario is depicted, except that ends of nonhomologous chromosomes are in the SUN-1 aggregate (violet loops). After fusion, chromosome ends will be shuffled. However, as the cytoplasmic forces overcome the attempt to synapse, one SUN-1 focus will be driven out of the patch. This tension-generated splitting event is one of the factors leading to the formation of high speed in the distribution of SUN-1 aggregates. Our data support other factors as sources for the high speed aggregates: recombination intermediates (C) and chromosomes entanglements (D).

We propose that duplets/multiplets of chromosome ends are linked to SUN-1 patches. These duplets/multiplets of chromosome ends attached to SUN-1 patches continue to move and meet other SUN-1 patches or foci. While the newly met SUN-1 aggregates coalesce, homology is assessed; how homology is assessed is still an open question. Chromosome ends are then shuffled through patches of SUN-1, and when the right partner is met, synapsis can take place. The shuffling of chromosome ends through the patches continues until all of the chromosomes have found their homolog. This process is dynamic, as most SUN-1 aggregates coalesce for less than 1 min. The shuffling of the chromosome ends during SUN-1 aggregate coalescence is, thus, one of the driving forces for homology search (Figure 8A and 8B). This allows homologous chromosome ends to meet and nonhomologous chromosomes to separate.

Chromosome ends coalescing in SUN-1 patches might not solely reflect shuffling chromosome ends but also interactions caused by recombination repair intermediates. Indeed, in *spo-11* fewer patches are formed than in wild type and introduction of DSBs significantly increases the formation of patches. These two processes (assessing homology) and ongoing repair of DSBs (up to the strand invasion step) might be the source of the long tails observed in the speed distribution of SUN-1 aggregates (see below). In addition interlocked chromosomes could contribute to the long tails.

### Formation of high-speed SUN-1 aggregates

During the leptotene/zygotene stage, SUN-1 aggregates in *C. elegans* moved more slowly and did not accelerate abruptly, similar to what was reported for telomere ends in budding yeast and maize [6], [7], [9]. Nonetheless, SUN-1 aggregates reached relatively high speeds (>160 nm/s), as demonstrated by the long tails in the distribution of the projected speed of SUN-1 aggregates.

Two nonexclusive explanations could account for the formation of the long tails in the projected speed distribution of SUN-1 aggregates: first, “a model of tension” [29], where assessment of homology (Figure 8A and 8B), a nascent repair intermediate (Figure 8C) or chromosome entanglements (Figure 8D) leads to the formation of tension between chromosome ends. These interactions are counteracted by cytoplasmic forces (generation of tension). When cytoplasmic forces overcome these interactions, the two chromosome ends are disjoined (loss of accumulated tension), leading to an increase in the speed distribution of SUN-1 aggregates. Absence of DSBs significantly reduces the formation of long tails. Indeed other factors, such as chromosome interlocks, could contribute to the formation of the tension. Recently, the repair protein *mlh-1* in *S. macrospora* had been assigned a critical role in resolution of interlocks [42]. When SYP-1 is depleted in *htp-1*, long tails reappear in the speed distribution despite their absence in *htp-1*, and fewer DSBs are made, as previously observed in *htp-1*;*syp-2* [21]. This suggests that entanglements can also contribute to the generation of tensions.

A second explanation for the formation of high speed could be that a patch containing paired homologs could move faster than a patch of nonpaired homologs. In the mutant SUN-1(S12E), pairing is fairly effective in the proximal part of the TZ [28]. The distribution of the projected speed of SUN-1 aggregates in this area was significantly shifted towards higher speeds (16% of long tails), supporting the idea that patches containing paired homologs could move faster (see Text S1, Videos S18 and S19, and Figures S7, S8, and S9). This is in contradiction with our finding that SUN-1 dynamics remained unchanged during the leptotene/zygotene stage despite processing of pairing. In fact, paired homologs in patches cannot account for the shift towards higher speeds, because we showed that patches (paired or unpaired homologs) and foci (most likely single chromosome ends) were both able to reach high speeds (Text S1). Patches are only faster than foci in the middle segment of the speed distribution. Addressing this paradox will require live imaging of the tagged end of a single chromosome.

### Exit of the polarized conformation

It has been shown that an unpaired chromosome keeps chromatin loosely clustered once stable strand invasion is established [43]. Similarly, we found that in *htp-1*; *syp-1*, chromatin was also loosely clustered concomitant with circular displacement tracks. Circular displacement tracks are likewise found in *cra-1* where partial synapsis is coupled with accumulation of DSB intermediates [34]. The fact that in *htp-1*; *syp-2* DSBs do not accumulate [21] could be an explanation why circular tracks were less frequent than in *cra-1*. Previously, we proposed that exit from polarized chromatin requires a certain recombination intermediate and/or full synapsis [28]. Loose chromatin clustering in *spo-11*, together with circular displacement tracks, could be explained by the absence of the particular recombination intermediate required to dissolve chromosomal attachment plaques.

### Chromosome end-led prophase movement alone is not sufficient for successful pairing

The absence of SUN-1 aggregates in *chk-2(me64*) confirms that this gene plays an indispensible role in pairing [28]. The triggers to activate CHK-2 are unknown, but DSBs could be one such trigger, as shown by the analyses of SUN-1 aggregates in aged *him-19(jf6)* irradiated worms [28]. In the present study, γ-irradiation significantly rescued some aspects of the behavior of SUN-1 aggregates in aged *him-19(jf6)*.

Despite their different roles during meiosis, *htp-1* and *prom-1* mutants display nonhomologous synapsis [20], [21], [32]. Deletion of either of these two proteins results in different phenotypes (precocious synapsis or delayed progression of meiosis). These mutants highlight the necessity of coordinating sub-events right after meiotic entry for the generation of functional SUN-1 aggregates. In both mutants, the speed distributions of SUN-1 aggregates lacked long tails; perhaps when chromosomes nonhomologously synapse, the tension-generating process cannot take place (assessment of pairing). The maximum number of aggregates was five in both cases, because when aggregates coalesce into a patch, chromosome ends could not be shuffled, because synapsis had already taken place. The two mutants were also similar in terms of the number of fusion/splitting events and of periodicity of coalescence time. The analysis of these two mutants strongly suggests that effective pairing of homologs requires more than just chromosome end movement. Other prophase I events are also necessary for successful pairing.

In this study, we showed that instead of single chromosome ends looking for their homologous partner, duplets or multiplets and single chromosome ends were brought together in groups (patches) during the leptotene/zygotene stage by SUN-1 aggregate movement. Chromosome ends shuffled through these patches in search of the correct partner. Simultaneous in vivo imaging of specific chromosome ends and SUN-1 aggregates should be the next step to substantiate our finding. Second, these patches might represent ongoing repair of DSBs. Deciphering the regulation of synapsis initiation while chromosome ends are being shuffled in a tightly regulated process is one of the challenges for the future.

## Materials and Methods

### Nematode strains, strain construction, and culture conditions

All *C. elegans* strains were cultured using standard techniques [44]. The following *C. elegans* strains were used: N2 Bristol, *sun-1(ok1282)*; *sun-1::GFP* [28], *sun-1(ok1282)*/nT1[*let-?qIs51*]; *sun-1::GFP(G311V)* [28], *chk-2(me64)*/*unc-51(e369) rol-9(sc148)*; *sun-1::GFP* [28], *syp-2(ok307)*/nT1[qIs51]; *sun-1::GFP* [28], *him-3(gk149)*/nT1[*let-?qIs51*]; *sun-1::GFP* [28], *htp-1(gk174)*/nT1[*let-?qIs51*]; *sun-1::GFP* [28] *him-19(jf6)*; *sun-1::GFP* [33], *syp-3(me42)*/hT2[*bli-4(e937)let-?(q782)qIs48*]; *sun-1::GFP*, *prom-1(ok1140)*; *sun-1::GFP*, *cra-1(tm2144)*/hT2(GFP); *sun-1::GFP*, *spo-11(me44)/*nT1[*unc-?(n754) let-? qIs50*]; *sun-1::GFP* [45].

Nematode strains were provided by the *Caenorhabditis Genetics Center*, which is funded by the NIH National Center for Research Resources (NCRR).

*him-19(jf6)*; *sun-1::GFP* and *sun-1(ok1282)*; *sun-1::GFP* worms were γ-irradiated with a dose of 50 Gy for 10 sec using a ^137^Cs source and the time lapse microscopy was recorded 2 hrs after irradiation. *syp-1(RNAi)* was done as described in [46].

### Time-lapse microscopy

For time lapse acquisitions, adult hermaphrodites preselected at the L4 stage 16 hrs before were mounted in a drop of 10 mM Levamisol on a 2% agarose pad and covered with a coverslip. For *him-19(jf6)*, worms were preselected 2 days before irradiation. The coverslip was sealed with melted Vaseline. Images were acquired at room temperature every 5 sec over a 15 min time period as stacks of optical sections at 1 µm intervals using a Deltavision deconvolution microscopy system (Applied Precision, Inc, 1040 12th Avenue, Northwest Issaquah, Washington 98027, USA) under the following conditions: FITC channel, 32%ND, bin 1×1, exposure time 200 msec, objective 60x. To assay the effect of Levamisole on the worms, worms were mounted as described above in a drop of Levamisole or M9 and then the hatch rate of the filmed worms evaluated under the two conditions. No significant decrease of the hatch rate was observed (Levamisol hatch rate: 93% (>500 eggs laid), M9 hatch rate: 96% (>200 eggs laid), Fisher's exact test: p>0.05).

Maximum intensity projection was done using Softworx software (Applied Precision, Inc, 1040 12th Avenue, Northwest Issaquah, Washington 98027, USA) and the collection of files saved. The collection of pictures was opened with Metamorph Offline (Molecular Devices, 402 Boot Rd., Downingtown, PA 19335, USA) and saved as stack files. To support the plotting process, the background of the stack pictures was removed using Autoquant X2 (AutoQuant Imaging, Troy, NY, USA) and the slices realigned using the first slice as a reference.

### Image analysis

With the help of Metamorph Offline, the positions of SUN-1 aggregates were manually followed. When two aggregates split, instead of starting to record two new tracks, tracking of the larger aggregate was continued. Then movement of the second aggregate was recorded. Subsequently positions of SUN-1 aggregates were plotted using Gnuplot software (Thomas Williams, Colin Kelley et al. 2004, http://www.gnuplot.info). Frequency and number of fusion/splitting events were computed using the number of aggregates as a function of time. Automated plotting of the movement of SUN-1 aggregates was done using Image J (NIH, http://rsbweb.nih.gov/ij/) and the plug-in MTrack2 (author: Nico Stuurman, http://valelab.ucsf.edu/people/p-stuurman.htm) (see Figure S9).

### Computations and statistics

The speed of the object between two successive data points was calculated as the distance covered divided by the time required to cover the distance. Arcs were computed as described in Figure S3. Three-dimensional reconstruction of SUN-1 aggregate movement was done using the relationship x^2^+y^2^+z^2^ = r^2^, where x, y, z are the coordinates of a point situated on a sphere of radius r. The z-coordinates were computed from the x- and y-coordinates of the nuclei viewed from the top as follows: z = √(r^2^−x^2^−y^2^), and when x^2^+y^2^>r^2^, the previous z-coordinate was used. Statistical analysis was done using the software R (R Development Core Team, http://www.R-project.org).

## Supporting Information

Figure S1 Background movement during time-lapse microscopy and definition of Maxwellian-shaped distribution. (A) Worms were killed in sodium azide and analyzed. Each line corresponds to the distribution of the projected speed of SUN-1::GFP aggregates in wild-type (blue), *him-3(gk149)* (yellow), and *htp-1(gk174)* (red) backgrounds. The corresponding cumulative projections of moving SUN-1::GFP aggregates are shown. (B) Left, a normal distribution, which is symmetric around the mean value. Right, a Maxwellian distribution with tails in the distribution (in red).
(0.89 MB TIF)

Figure S2 Arc computation. Distances *a* and *b* were calculated from the most extreme positions of the tracks (orange in A [SUN-1::GFP], red in A' [SUN-1(G311V)::GFP]). Distance *c* was calculated using the Pythagoras theorem (B, B'). *c* was circumscribed on a circle with the radius of the average size of a nucleus. Angle β (pink) was calculated using the cosine law (C, C'). When the distance *c* was greater than the radius *r* (D), the value of *r* was increased so that *c* could be circumscribed and the line for this arc is shown as a dotted line (e.g., Figure 4D). Description of the tracks by expressing the sum of their total length is misleading, because this does not reflect how far the tracks reach. For example, small oscillations, as in SUN-1(G311V), add up to large distances traveled, although the aggregates have not moved far. Arcs have the advantage of allowing for comparisons between different genotypes (compare C and C').
(0.20 MB TIF)

Figure S3 Lack of patterns for the traveled distance of SUN-1::GFP aggregates in nuclei located at different positions in the TZ. Box plot of the arc values for subregions in TZ (distal, central, and proximal parts of TZ). Red box plot, first movie; blue box plot, second movie. Green line represents the median value in the distribution of the arc; extremities of the whiskers are minima and maxima; bottom of the box, first quartile; top of the box, last quartile of the distribution of the arcs.
(0.27 MB TIF)

Figure S4 Lateral elements are required for proper loading of PC proteins. Localization of the PC protein ZIM-3 in *him-3(gk149)* and *htp-1(gk174)*. Immunostaining of ZIM-3 (red) and SUN-1::GFP (green); DAPI (blue).
(1.96 MB TIF)

Figure S5 SUN-1 aggregates are present in nuclei with loose clustering of the chromatin. Immunostaining of SUN-1 in wild type, *htp-1(gk174)*; *syp-1(RNAi)* and *cra-1(tm2144)*. White arrows indicate nuclei with loose clustering of the chromatin.
(3.82 MB TIF)

Figure S6 An excess of DSBs affected pairing but not SC polymerization. Dissected gonads of aged irradiated and nonirradiated worms were divided into seven zones of equal length (A), and the pairing of homologs was assessed by FISH with a probe for 5S rDNA (on chromosome V). (B) Pairing in nonirradiated 3-d-old wild-type gonads (dark blue) and three-d-old wild-type gonads 24 h after irradiation (light blue). The histogram shows at least two gonads with or without irradiation. Asterisks highlight the differences that are significant (Fisher's exact test, *p*<0.05). (C) SYP-1 polymerization in wild-type worms without irradiation (upper part) and γ-irradiated wild-type worms 2 h after irradiation (lower part).
(3.05 MB TIF)

Figure S7 Effect of SUN-1 phosphorylation on aggregate dynamics. Projection of the cumulative movement of SUN-1(S12E)::GFP (A, A'), displacement tracks (B, B'), distribution of the projected speed (C, C'), and arcs (D, D'). Blue lines represent values from the first movie; orange lines values from the second. (A, B, C, D) from distal TZ, (A', B', C', D') from proximal TZ. See Figure S8 for number of nuclei analyzed. Scale bar: 2 µm.
(1.46 MB TIF)

Figure S8 Dynamics of SUN-1(S12E)::GFP aggregates. (A) Numbers of aggregates in the regions proximal to the mitotic zone. The variations indicated correspond to the standard deviation. (B) Number of SUN-1 fusion/splitting events grouped into classes. (C) Quantification of the coalescence time (*t*) grouped into classes (*t*<1 min, 1 min≤*t*<3 min, and *t*≥3 min). (D) Fisher's exact test to assess the difference between wild type and SUN-1(S12E)::GFP for the values ‘number of fusion/splitting events’. (E) Fisher's exact to assess the difference between wild type and SUN-1(S12E)::GFP for the values ‘time-window of SUN-1 aggregate coalescence’.
(0.47 MB TIF)

Figure S9 Correlation between speed and size of SUN-1 aggregates. (A) A deconvolved image was converted to a binary image using a threshold and then initial segmentation restored using a watershed transform (i). Aggregates <2.15 µm^2^ were defined as foci, aggregates >2.15 µm^2^ as patches. Output image (yellow) overlaid with the starting picture (i, right panel). Collection of background-subtracted movies treated the same manner (ii). Correlation factor  = −0.017 for size of foci and their speed. Correlation factor  = −0.016 for size of patches and their speed. No correlation was found in either case. (B) CDF of the foci (red) and patches (green). Differences in distribution of the projected speed of SUN-1 foci and patches are highlighted by the subtraction of the CDF of the foci to the CDF of the patches (blue). SUN-1 patches in the range of 15–120 nm/s moved faster than SUN-1 foci.
(0.92 MB TIF)

Table S1 Fisher's exact test to assess the difference between wild type and all other genotypes tested for the values ‘number of fusion/splitting events’. Significant p-values (p<0.05) are highlighted in bold.
(0.06 MB DOC)

Table S2 Fisher's exact to assess the difference between wild type and all other genotypes tested for the values' time-window of SUN-1 aggregate coalescence'. Significant p-values (p<0.05) are highlighted in bold.
(0.05 MB DOC)

Text S1 Supplemental results.
(0.04 MB DOC)

Text S2 Supplemental methods.
(0.03 MB DOC)

Video S1 Time lapse series of SUN-1::GFP with counterstaining of the chromatin using Hoechst 33342. The first inset shows the merging of SUN-1::GFP (green) and the chromatin (blue). The second inset shows the chromatin and the third inset SUN-1::GFP. Scale bar: 2 µm.
(1.16 MB MOV)

Video S2 Time lapse series of SUN-1::GFP in the *sun-1(ok1282)* background. The three insets show three enlarged independent nuclei. Scale bar: 2 µm.
(0.76 MB MOV)

Video S3 Time lapse series of SUN-1(G311V)::GFP in the *sun-1(ok1282)* background. The three insets show three enlarged independent nuclei. Scale bar: 2 µm.
(0.84 MB MOV)

Video S4 Time lapse series of SUN-1::GFP in the *him-3(gk149)* background. The three insets show three enlarged independent nuclei. Scale bar: 2 µm.
(0.56 MB MOV)

Video S5 Time lapse series of SUN-1::GFP in the *htp-1(gk174)* background. The three insets show three enlarged independent nuclei. Scale bar: 2 µm.
(0.26 MB MOV)

Video S6 Time lapse series of SUN-1::GFP in the *syp-2(ok307)* background; distal part of the TZ. The three insets show three enlarged independent nuclei. Scale bar: 2 µm.
(0.55 MB MOV)

Video S7 Time lapse series of SUN-1::GFP in the *syp-2(ok307)* background; proximal part of the TZ. The three insets show three enlarged independent nuclei. Scale bar: 2 µm.
(0.55 MB MOV)

Video S8 Time lapse series of SUN-1::GFP in the *syp-3(me42)* background. The three insets show three enlarged independent nuclei. Scale bar: 2 µm.
(0.55 MB MOV)

Video S9 Time lapse series of SUN-1::GFP in the *htp-1(gk174)*; *syp-1(RNAi)* background in the distal part of the zone with SUN-1 aggregates. The three insets show three enlarged independent nuclei. Scale bar: 2 µm.
(0.87 MB MOV)

Video S10 Time lapse series of SUN-1::GFP in the *htp-1(gk174)*; *syp-1(RNAi)* background in the proximal part of the zone with SUN-1 aggregates. The three insets show three enlarged independent nuclei. Scale bar: 2 µm.
(0.84 MB MOV)

Video S11 Time lapse series of SUN-1::GFP in the *prom-1(ok1140)* background. The three insets show three enlarged independent nuclei. Scale bar: 2 µm.
(0.84 MB MOV)

Video S12 Time lapse series of SUN-1::GFP in the *him-19(jf6*) background; non-irradiated 2-d-old hermaphrodites. Scale bar: 2 µm.
(0.96 MB MOV)

Video S13 Time lapse series of SUN-1::GFP in the *him-19(jf6*) background; irradiated 2-d-old hermaphrodites. The three insets show three enlarged independent nuclei. Scale bar: 2 µm.
(0.84 MB MOV)

Video S14 Time lapse series of SUN-1::GFP in the *sun-1(ok1282)* background; irradiated 2-d-old hermaphrodites. The three insets show three enlarged independent nuclei. Scale bar: 2 µm.
(0.84 MB MOV)

Video S15 Time lapse series of SUN-1::GFP in the *cra-1(tm2144)* background. The three insets show three enlarged independent nuclei. Scale bar: 2 µm.
(0.98 MB MOV)

Video S16 Time lapse series of SUN-1::GFP in the *spo-11(me44)* background. The three insets show three enlarged independent nuclei. Scale bar: 2 µm.
(0.84 MB MOV)

Video S17 Time lapse series of SUN-1::GFP in the *spo-11(me44)* background; irradiated 2-d-old hermaphrodites. The three insets show three enlarged independent nuclei. Scale bar: 2 µm.
(0.84 MB MOV)

Video S18 Time lapse series of SUN-1(S12E)::GFP in the *sun-1(ok1282)* background in the distal part of the prolonged TZ. The three insets show three enlarged independent nuclei. Scale bar: 2 µm.
(0.98 MB MOV)

Video S19 Time lapse series of SUN-1(S12E)::GFP in the *sun-1(ok1282)* background in the proximal part of the prolonged TZ. The three insets show three enlarged independent nuclei. Scale bar: 2 µm.
(0.98 MB MOV)
