# Supplementary figures and images for: Leptotene/Zygotene Chromosome Movement Via the SUN/KASH Protein Bridge in Caenorhabditis elegans
Source: PLoS Genet. 2010 Nov 24;6(11):e1001219. doi: 10.1371/journal.pgen.1001219 (PMC2991264; doi:10.1371/journal.pgen.1001219)

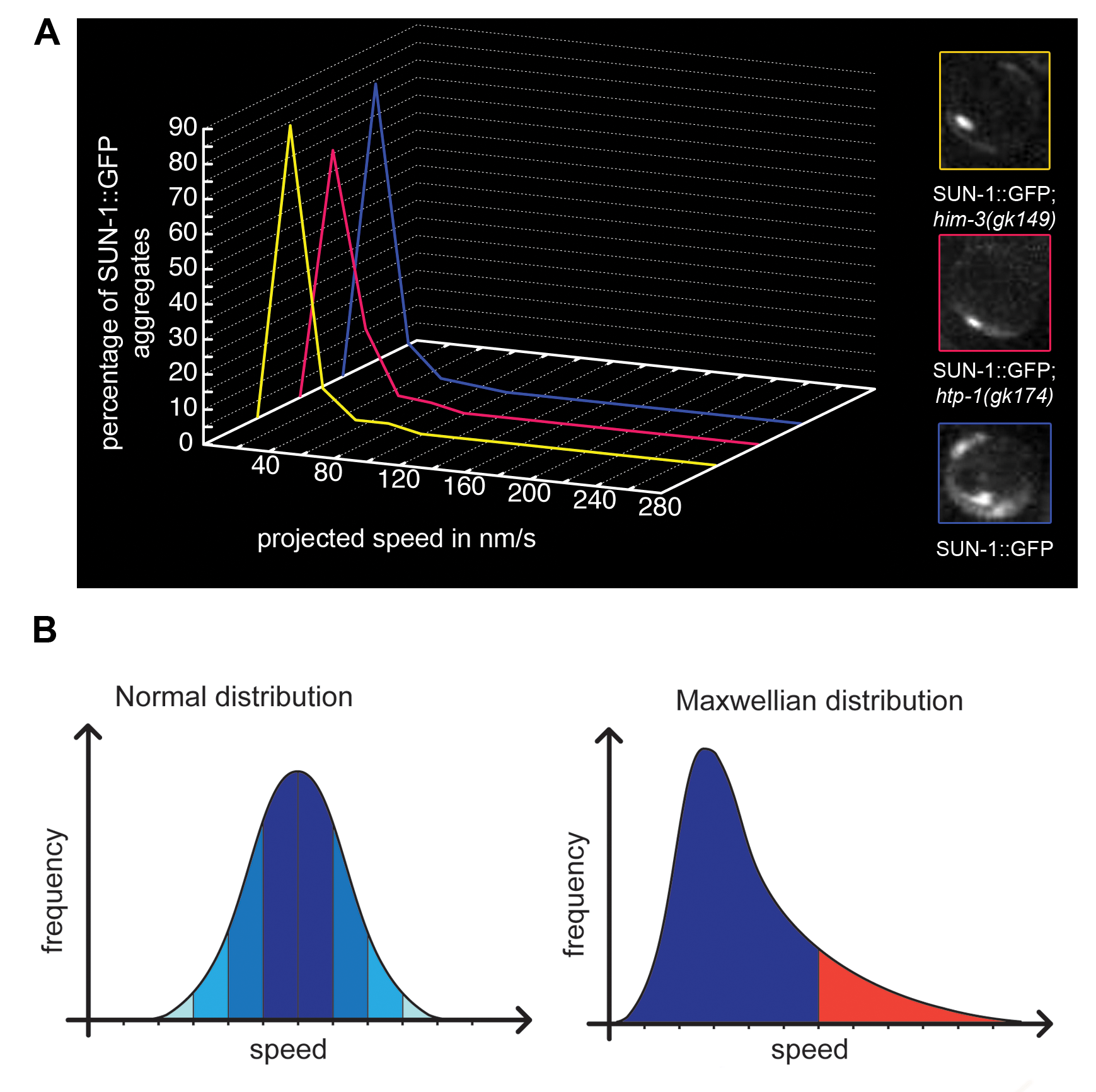

Supplement: Figure S1 — Background movement during time-lapse microscopy and definition of Maxwellian-shaped distribution. (A) Worms were killed in sodium azide and analyzed. Each line corresponds to the distribution of the projected speed of SUN-1::GFP aggregates in wild-type (blue), him-3(gk149) (yellow), and htp-1(gk174) (red) backgrounds. The corresponding cumulative projections of moving SUN-1::GFP aggregates are shown. (B) Left, a normal distribution, which is symmetric around the mean value. Right, a Maxwellian distribution with tails in the distribution (in red). (0.89 MB TIF) [file pgen.1001219.s001.tif]

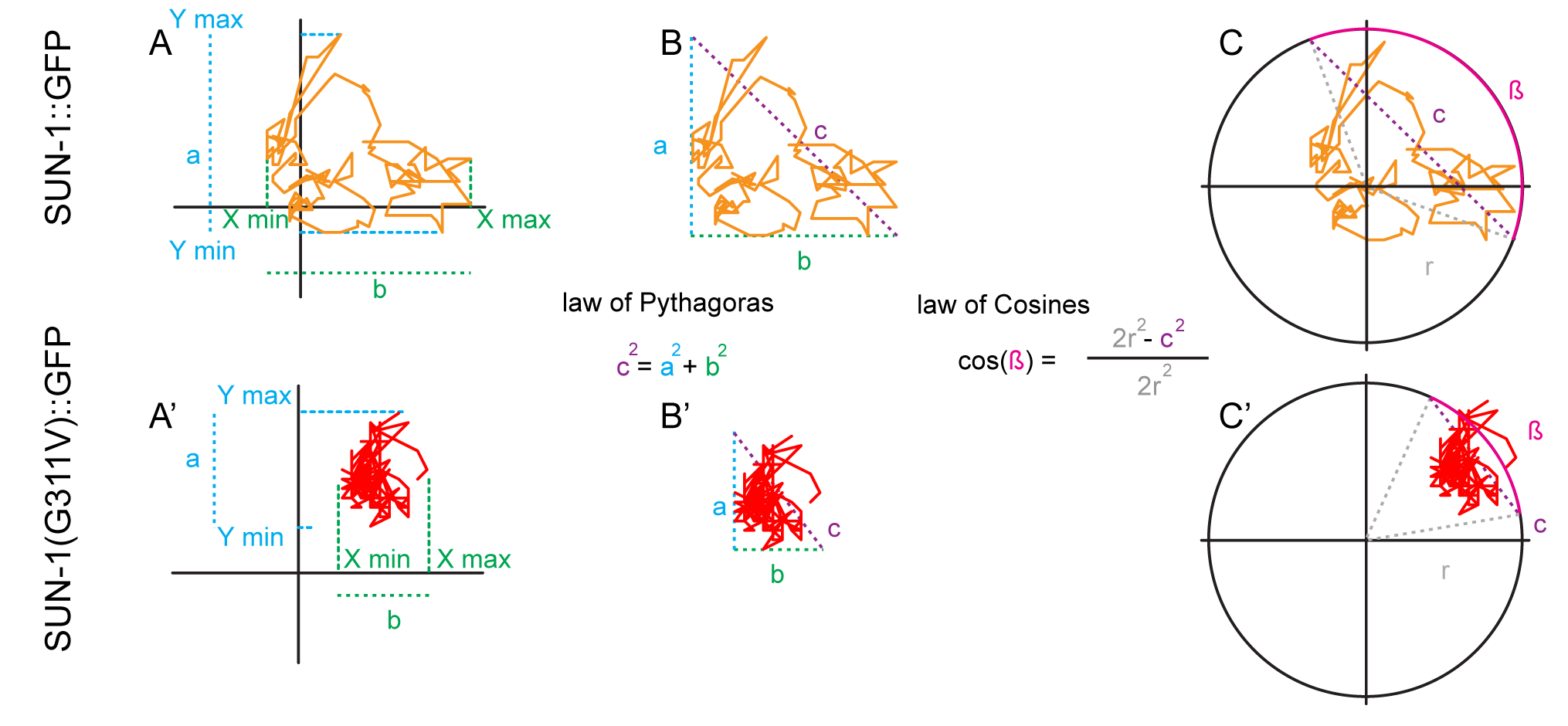

Supplement: Figure S2 — Arc computation. Distances a and b were calculated from the most extreme positions of the tracks (orange in A [SUN-1::GFP], red in A' [SUN-1(G311V)::GFP]). Distance c was calculated using the Pythagoras theorem (B, B'). c was circumscribed on a circle with the radius of the average size of a nucleus. Angle β (pink) was calculated using the cosine law (C, C'). When the distance c was greater than the radius r (D), the value of r was increased so that c could be circumscribed and the line for this arc is shown as a dotted line (e.g., Figure 4D). Description of the tracks by expressing the sum of their total length is misleading, because this does not reflect how far the tracks reach. For example, small oscillations, as in SUN-1(G311V), add up to large distances traveled, although the aggregates have not moved far. Arcs have the advantage of allowing for comparisons between different genotypes (compare C and C'). (0.20 MB TIF) [file pgen.1001219.s002.tif]

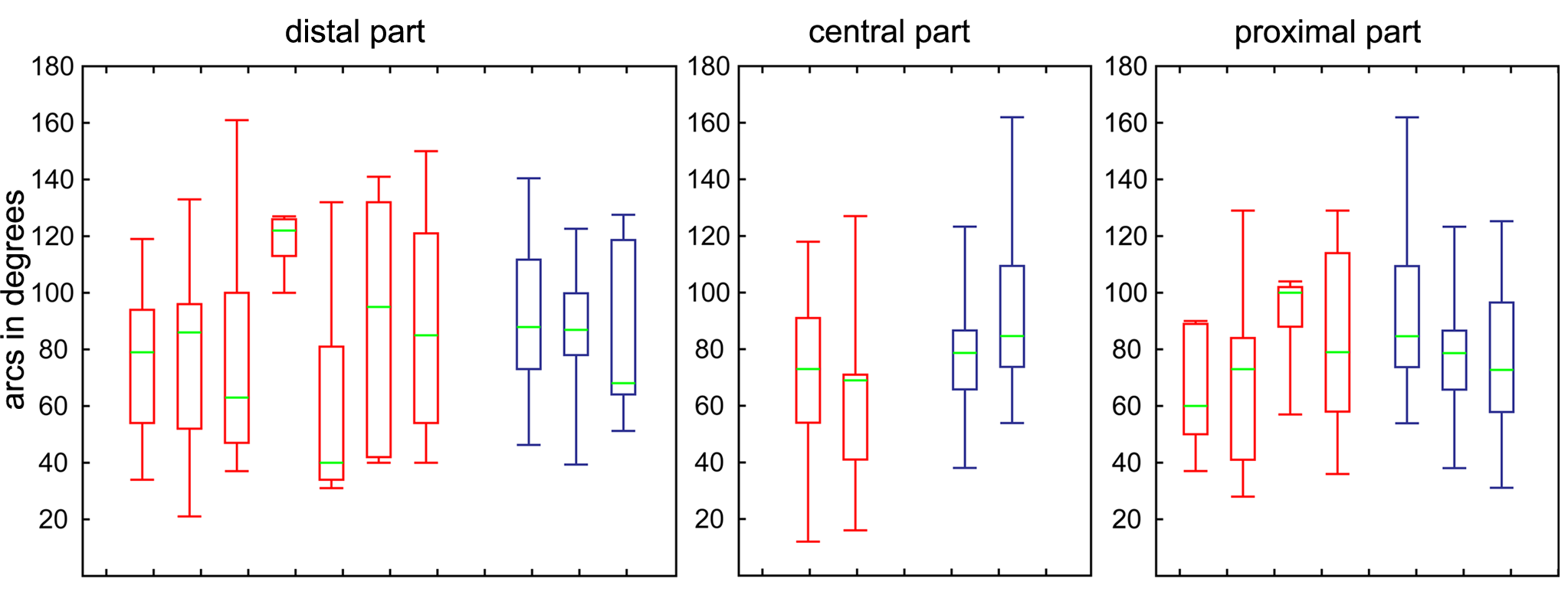

Supplement: Figure S3 — Lack of patterns for the traveled distance of SUN-1::GFP aggregates in nuclei located at different positions in the TZ. Box plot of the arc values for subregions in TZ (distal, central, and proximal parts of TZ). Red box plot, first movie; blue box plot, second movie. Green line represents the median value in the distribution of the arc; extremities of the whiskers are minima and maxima; bottom of the box, first quartile; top of the box, last quartile of the distribution of the arcs. (0.27 MB TIF) [file pgen.1001219.s003.tif]

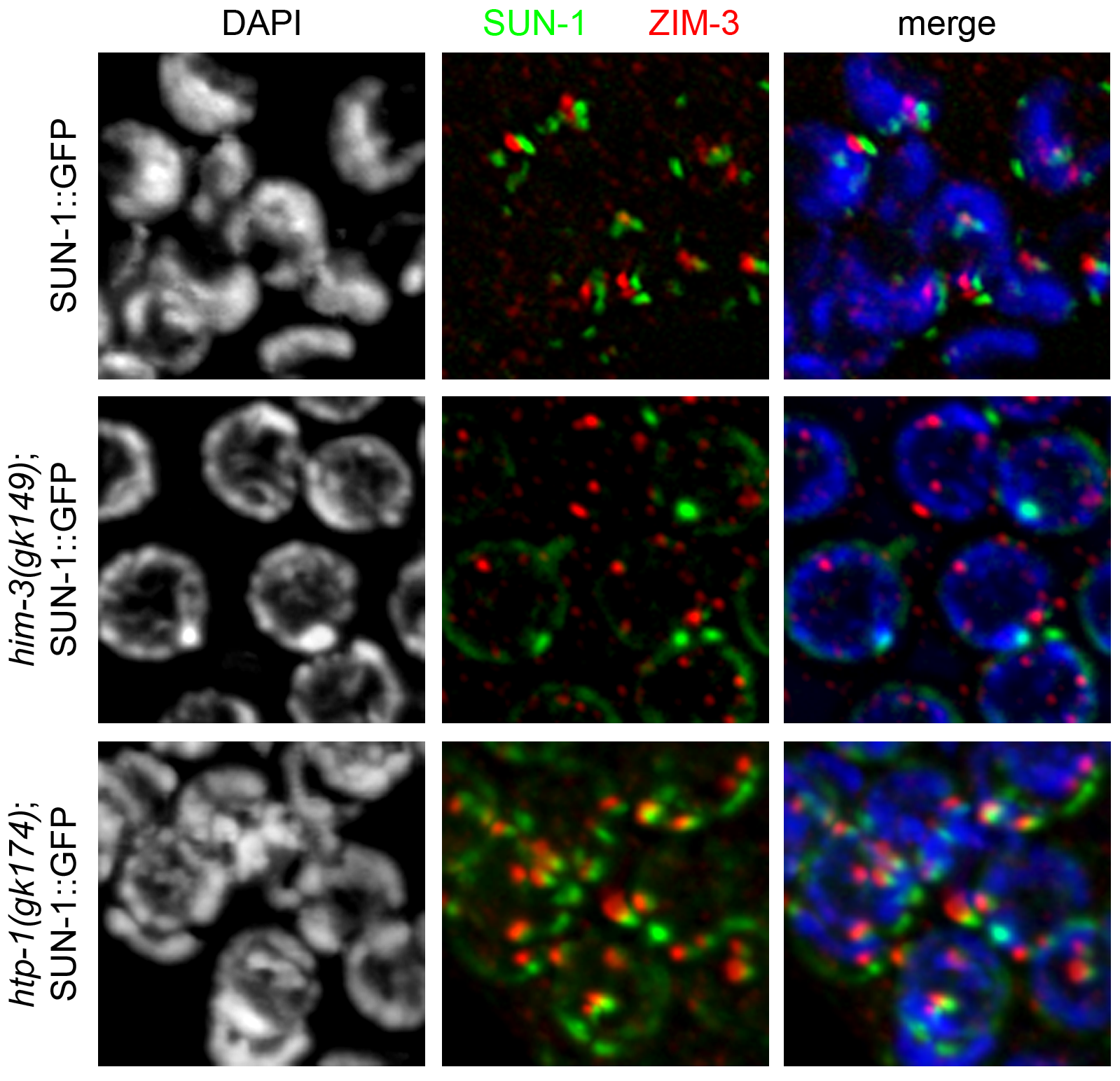

Supplement: Figure S4 — Lateral elements are required for proper loading of PC proteins. Localization of the PC protein ZIM-3 in him-3(gk149) and htp-1(gk174). Immunostaining of ZIM-3 (red) and SUN-1::GFP (green); DAPI (blue). (1.96 MB TIF) [file pgen.1001219.s004.tif]

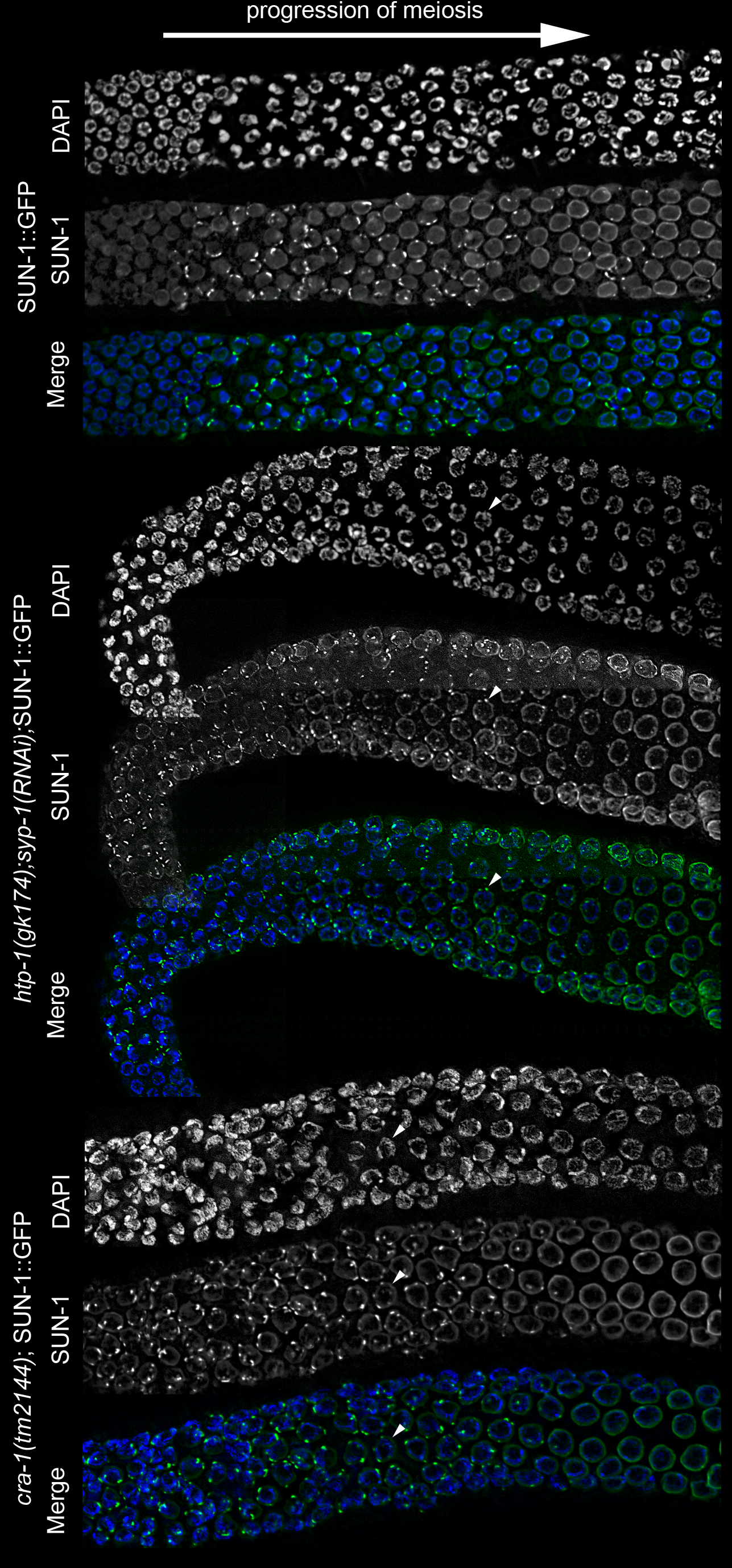

Supplement: Figure S5 — SUN-1 aggregates are present in nuclei with loose clustering of the chromatin. Immunostaining of SUN-1 in wild type, htp-1(gk174); syp-1(RNAi) and cra-1(tm2144). White arrows indicate nuclei with loose clustering of the chromatin. (3.82 MB TIF) [file pgen.1001219.s005.tif]

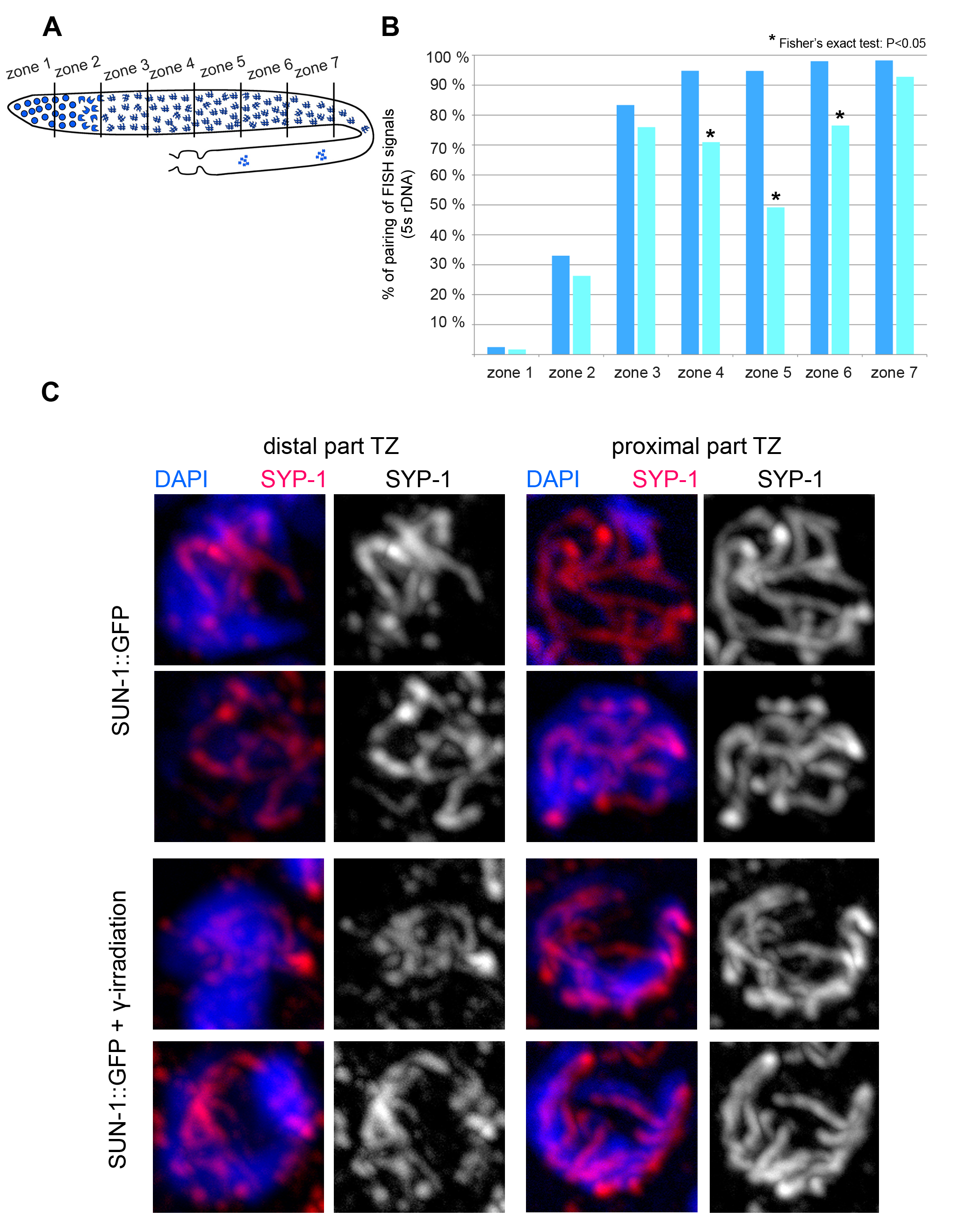

Supplement: Figure S6 — An excess of DSBs affected pairing but not SC polymerization. Dissected gonads of aged irradiated and nonirradiated worms were divided into seven zones of equal length (A), and the pairing of homologs was assessed by FISH with a probe for 5S rDNA (on chromosome V). (B) Pairing in nonirradiated 3-d-old wild-type gonads (dark blue) and three-d-old wild-type gonads 24 h after irradiation (light blue). The histogram shows at least two gonads with or without irradiation. Asterisks highlight the differences that are significant (Fisher's exact test, p<0.05). (C) SYP-1 polymerization in wild-type worms without irradiation (upper part) and γ-irradiated wild-type worms 2 h after irradiation (lower part). (3.05 MB TIF) [file pgen.1001219.s006.tif]

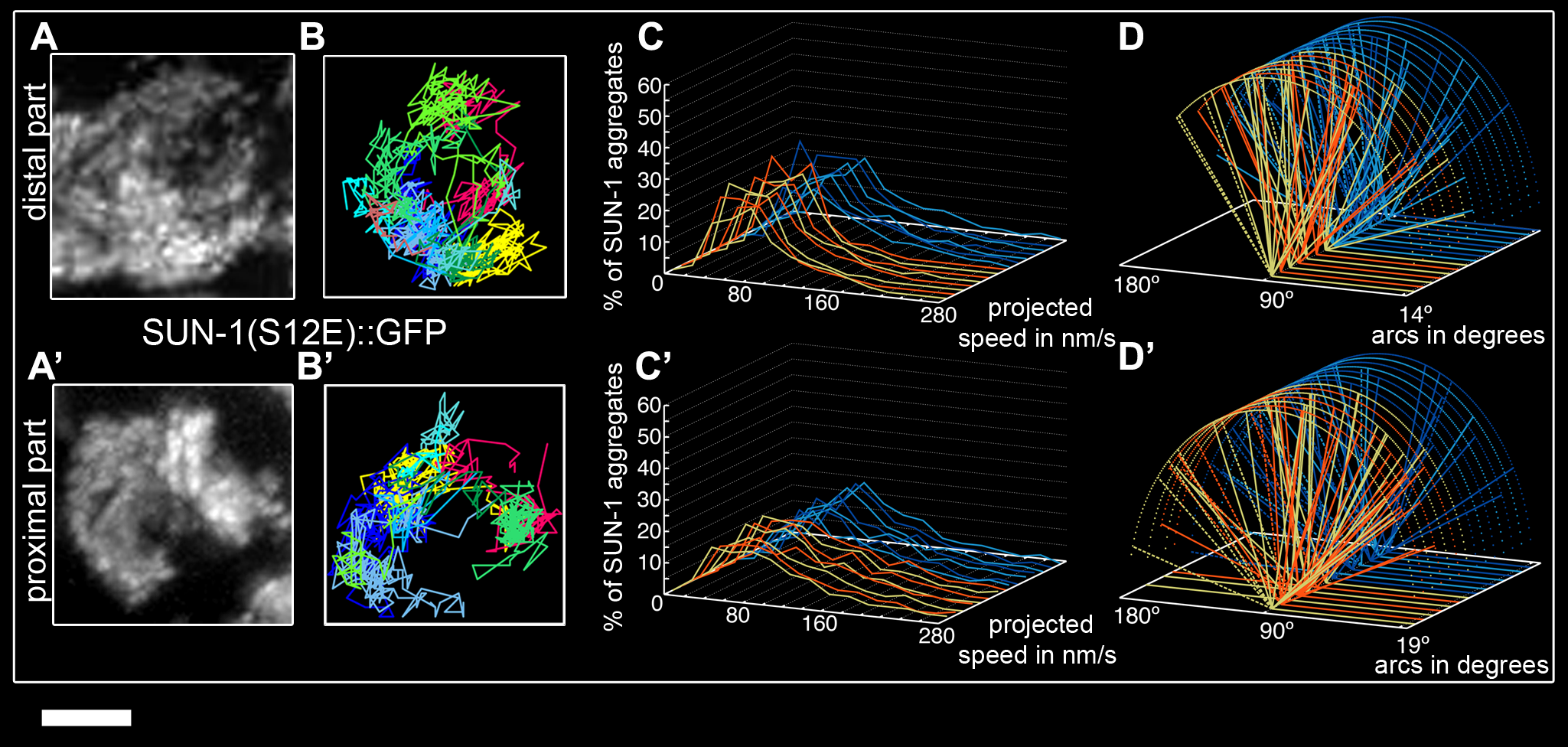

Supplement: Figure S7 — Effect of SUN-1 phosphorylation on aggregate dynamics. Projection of the cumulative movement of SUN-1(S12E)::GFP (A, A'), displacement tracks (B, B'), distribution of the projected speed (C, C'), and arcs (D, D'). Blue lines represent values from the first movie; orange lines values from the second. (A, B, C, D) from distal TZ, (A', B', C', D') from proximal TZ. See Figure S8 for number of nuclei analyzed. Scale bar: 2 µm. (1.46 MB TIF) [file pgen.1001219.s007.tif]

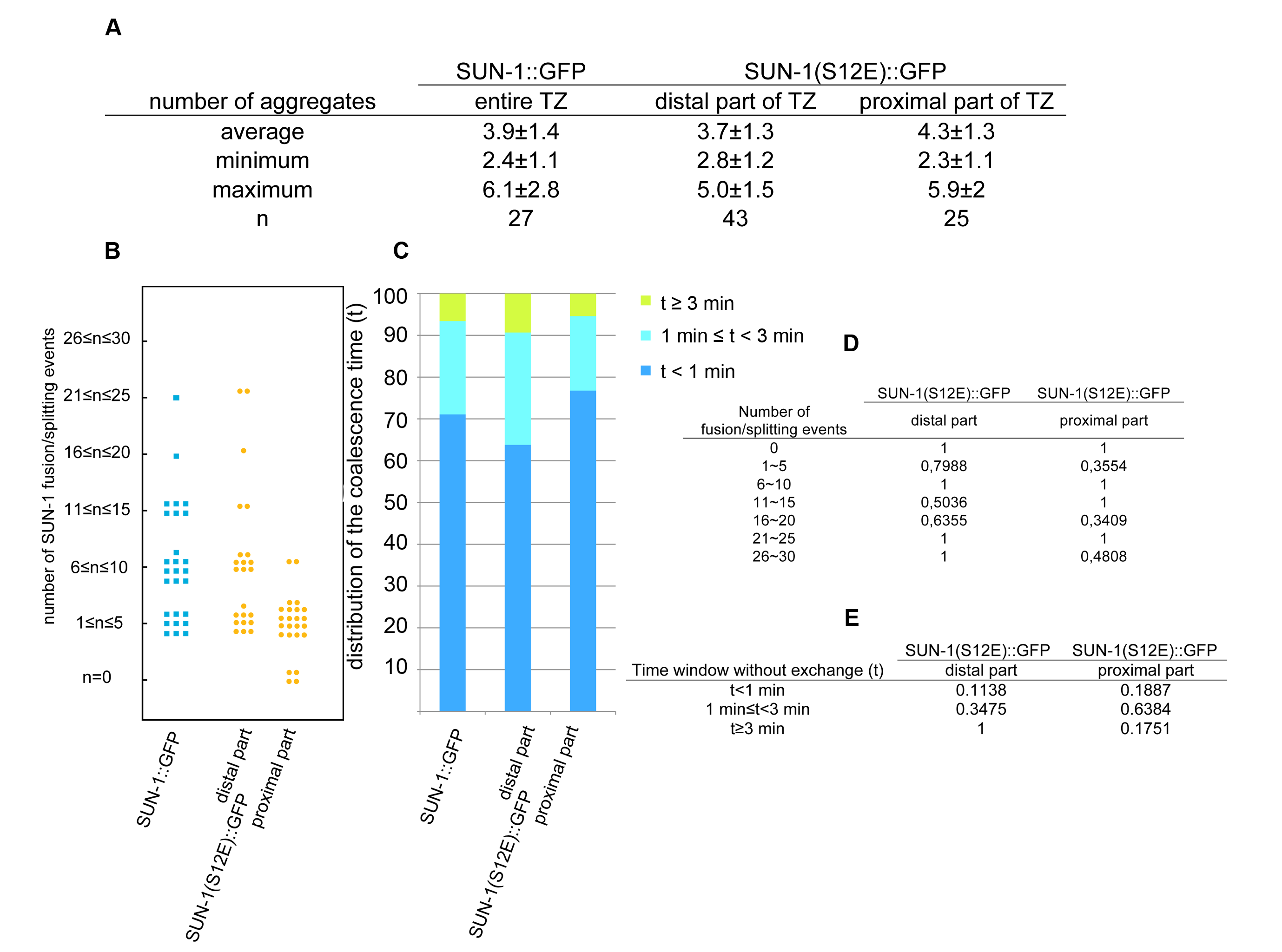

Supplement: Figure S8 — Dynamics of SUN-1(S12E)::GFP aggregates. (A) Numbers of aggregates in the regions proximal to the mitotic zone. The variations indicated correspond to the standard deviation. (B) Number of SUN-1 fusion/splitting events grouped into classes. (C) Quantification of the coalescence time (t) grouped into classes (t<1 min, 1 min≤t<3 min, and t≥3 min). (D) Fisher's exact test to assess the difference between wild type and SUN-1(S12E)::GFP for the values ‘number of fusion/splitting events’. (E) Fisher's exact to assess the difference between wild type and SUN-1(S12E)::GFP for the values ‘time-window of SUN-1 aggregate coalescence’. (0.47 MB TIF) [file pgen.1001219.s008.tif]

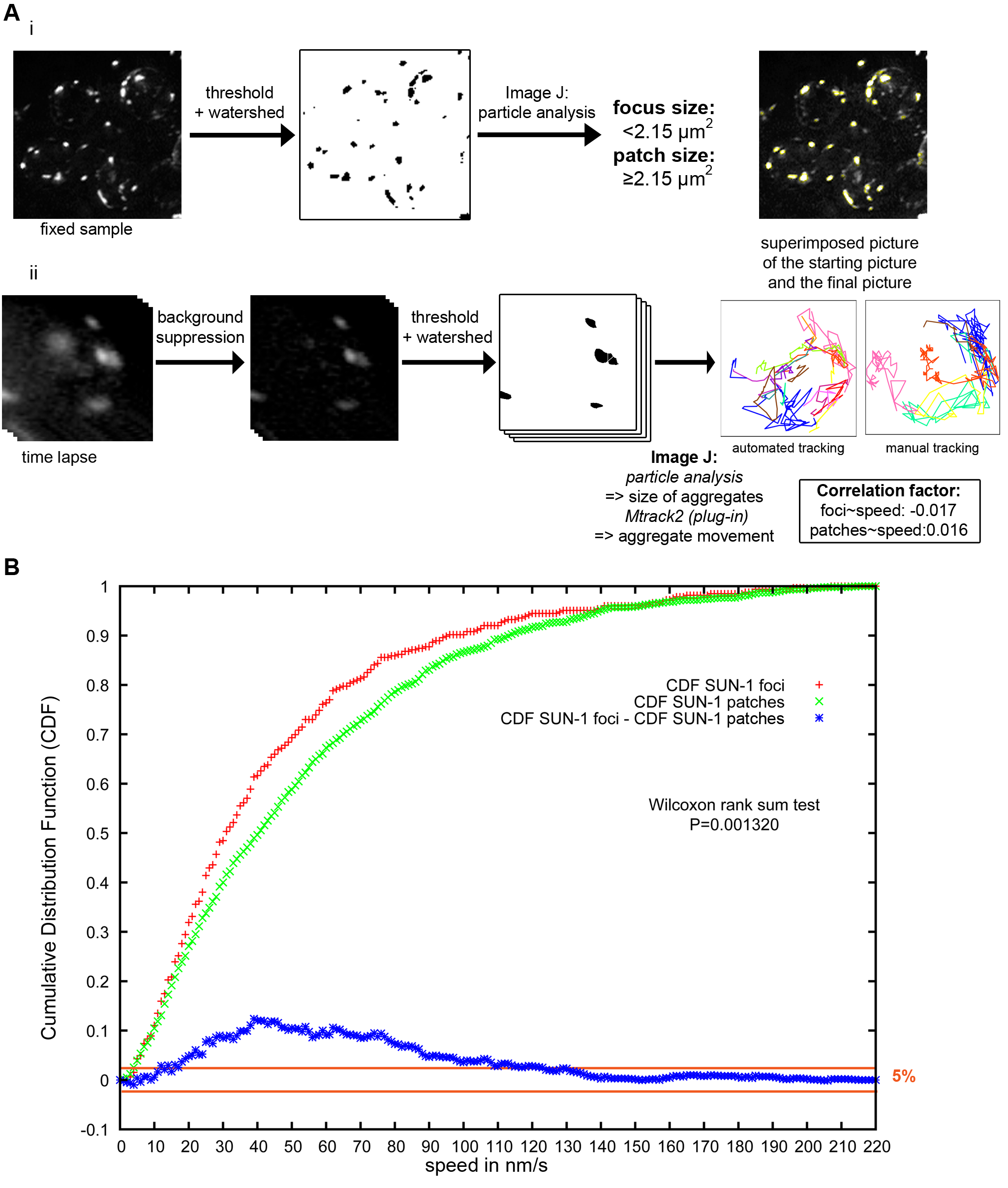

Supplement: Figure S9 — Correlation between speed and size of SUN-1 aggregates. (A) A deconvolved image was converted to a binary image using a threshold and then initial segmentation restored using a watershed transform (i). Aggregates <2.15 µm2 were defined as foci, aggregates >2.15 µm2 as patches. Output image (yellow) overlaid with the starting picture (i, right panel). Collection of background-subtracted movies treated the same manner (ii). Correlation factor = −0.017 for size of foci and their speed. Correlation factor = −0.016 for size of patches and their speed. No correlation was found in either case. (B) CDF of the foci (red) and patches (green). Differences in distribution of the projected speed of SUN-1 foci and patches are highlighted by the subtraction of the CDF of the foci to the CDF of the patches (blue). SUN-1 patches in the range of 15–120 nm/s moved faster than SUN-1 foci. (0.92 MB TIF) [file pgen.1001219.s009.tif]
