# Supplementary material for: Leptotene/Zygotene Chromosome Movement Via the SUN/KASH Protein Bridge in Caenorhabditis elegans
Source: PLoS Genet. 2010 Nov 24;6(11):e1001219. doi: 10.1371/journal.pgen.1001219 (PMC2991264; doi:10.1371/journal.pgen.1001219)
Supplement: Table S1 — Fisher's exact test to assess the difference between wild type and all other genotypes tested for the values ‘number of fusion/splitting events’. Significant p-values (p<0.05) are highlighted in bold. (0.06 MB DOC) [file pgen.1001219.s010.doc]

**Table S1. Fisher’s exact test to assess the difference between wild type and all other genotypes tested for the values ‘number of fusion/splitting events’.** Significant p-values (p<0.05) are highlighted in bold.

|  | SUN-1(G311V)::GFP | *him-3(gk149)*;  SUN-1::GFP | *htp-1(gk174)*;  SUN-1::GFP | *syp-2(ok307)*;  SUN-1::GFP | *syp-2(ok307)*;  SUN-1::GFP | *syp-3(me42)*;  SUN-1::GFP |
| --- | --- | --- | --- | --- | --- | --- |
| Number offusion/splitting events | entire TZ | entire TZ | entire TZ | distal part | proximal part | entire TZ |
| 0 | **4,249E-08** | **2,2E-16** | **0,04538** | 0,5023 | 0,1504 | 1 |
| 1~5 | 0,0815 | **0,00002905** | **0,03162** | **0,0002281** | 0,09493 | 0,3256 |
| 6~10 | 0,07339 | **0,00000769** | 0,158 | **0,04098** | 0,7998 | 1 |
| 11~15 | 0,2994 | **0,001248** | 0,06921 | **0,004357** | **0,008902** | 0,3898 |
| 16~20 | 1 | 0,3506 | 1 | 0,4286 | 0,3699 | 1 |
| 21~25 | 1 | 0,3506 | 1 | 0,4286 | 0,3699 | 1 |
| 26~30 | 1 | 1 | 1 | 1 | 1 | 1 |

|  | *htp-1(gk149); syp-1(RNAi);*  SUN-1::GFP | | *spo-11(me44)*;  SUN-1::GFP | *spo-11(me44)*; SUN-1:  :GFP irradiated |
| --- | --- | --- | --- | --- |
| Number of fusion/splitting events | distal part of TZ | proximal part of TZ | entire TZ | distal part of TZ |
| 0 | 1 | **0.04252** | 1 | 1 |
| 1~5 | 0.563 | **0.00473** | 0.1563 | 0.1119 |
| 6~10 | 1 | **0.02153** | 0.1376 | 1 |
| 11~15 | 0.2609 | **0.02391** | 1 | 0.2284 |
| 16~20 | 1 | 1 | 1 | 0.3223 |
| 21~25 | 0.5881 | 1 | 1 | 1 |
| 26~30 | 1 | 1 | 1 | 1 |

|  | *him-19(jf6)*; SUN-1::GFP | *him-19(jf6)*; SUN-1::GFP irradiated | SUN-1::GFP irradiated | *prom-1(ok1140)*; SUN-1::GFP | *cra-1(tm2144)*; SUN-1::GFP |
| --- | --- | --- | --- | --- | --- |
| Number of fusion/splitting events | entire TZ | entire TZ | entire TZ | entire TZ | entire TZ |
| 0 | 1 | **0.02804** | 0.4130 | **0,0001014** | 1 |
| 1~5 | 0.2667 | **0.03699** | 1 | **0,02854** | **0.01529** |
| 6~10 | 0.7115 | **0.03269** | 0.767 | 0,2023 | 0.3575 |
| 11~15 | 0.3026 | 0.1239 | 1 | **0,02292** | 0.1267 |
| 16~20 | 1 | 0.45 | 1 | 1 | 0.1597 |
| 21~25 | 1 | 0.45 | 1 | 1 | 0.5809 |
| 26~30 | 1 | 1 | 1 | 1 | 1 |
