# Supplementary material for: Leptotene/Zygotene Chromosome Movement Via the SUN/KASH Protein Bridge in Caenorhabditis elegans
Source: PLoS Genet. 2010 Nov 24;6(11):e1001219. doi: 10.1371/journal.pgen.1001219 (PMC2991264; doi:10.1371/journal.pgen.1001219)
Supplement: Table S2 — Fisher's exact to assess the difference between wild type and all other genotypes tested for the values' time-window of SUN-1 aggregate coalescence'. Significant p-values (p<0.05) are highlighted in bold. (0.05 MB DOC) [file pgen.1001219.s011.doc]

**Table S2. Fisher’s exact to assess the difference between wild type and all other genotypes tested for the values’ time-window of SUN-1 aggregate coalescence’.** Significant p-values (p<0.05) are highlighted in bold*.*

|  | *htp-1(gk174)*; SUN-1::GFP | *syp-2(ok307)*; SUN-1::GFP | *syp-2(ok307)*; SUN-1::GFP | *syp-3(me42)*; SUN-1::GFP |
| --- | --- | --- | --- | --- |
| Time window without exchange (t) | entire TZ | distal part | proximal part | entire TZ |
| t<1 min | **0.01260** | **4.24e-05** | 0.1457 | **2.906e-05** |
| 1 min≤t<3 min | 0.1172 | 0.27 | 1 | **0.03373** |
| t≥3 min | 1 | 0.05597 | 0.1617 | **0.0002778** |

|  | *htp-1(gk149); syp-1(RNAi);*  SUN-1::GFP | | *spo-11(me44)*;  SUN-1::GFP | *spo-11(me44)*; SUN-1:  :GFP irradiated |
| --- | --- | --- | --- | --- |
| Time window without exchange (t) | distal part of TZ | proximal part of TZ | entire TZ | distal part of TZ |
| t<1 min | 1 | **0.04252** | 0.09603 | **0.01331** |
| 1 min≤t<3 min | 0.563 | **0.00473** | 0.633 | **0.02071** |
| t≥3 min | 1 | **0.02153** | 0.2104 | 0.0777 |

|  | *him-19(jf6)*; SUN-1::GFP | *him-19(jf6)*; SUN-1::GFP irradiated | SUN-1::GFP irradiated | *prom-1(ok1140)*; SUN-1::GFP | *cra-1(tm2144)*; SUN-1::GFP |
| --- | --- | --- | --- | --- | --- |
| Time window without exchange (t) | entire TZ | entire TZ | entire TZ | entire TZ | entire TZ |
| t<1 min | **6.884e-05** | **7.537e-05** | **0.04290** | **0.0009864** | 0.2205 |
| 1 min≤t<3 min | **0.006124** | 0.394 | **0.000807** | 0.08724 | 0.1417 |
| t≥3 min | 0.2577 | **0.008372** | 1 | **5.776e-05** | 0.443 |
