# Supplementary material for: Leptotene/Zygotene Chromosome Movement Via the SUN/KASH Protein Bridge in Caenorhabditis elegans
Source: PLoS Genet. 2010 Nov 24;6(11):e1001219. doi: 10.1371/journal.pgen.1001219 (PMC2991264; doi:10.1371/journal.pgen.1001219)
Supplement: Text S1 — Supplemental results. (0.04 MB DOC) [file pgen.1001219.s012.doc]

**Text S1. Supplemental results.**

**Effect of phosphorylation state of SUN-1(S12) on SUN-1 aggregate dynamics**

Recently, we showed that the nuclear N-terminus of SUN-1 is subjected to multiple phosphorylations at the onset of leptotene/zygotene, when chromosome movement is observed. Phospho-mimicking substitutions of the phosphorylation sites revealed that they are involved in the regulation of aggregate dissolution and meiotic progression [1]. We subjected a transgenic line bearing the mutated phospho-target site S12E to time-lapse analysis.

In the gonads of hermaphrodite worms solely expressing SUN-1(S12E)::GFP, which mimics constitutive phosphorylation of S12, the zone of nuclei displaying a clustered conformation of chromatin was extended compared to wild type [1]. The aggregates covered a surface area resembling the crescent shape of the chromatin (Figure S7A, S7B, S7A′, and S7B′). Because the clustering of the chromatin was prolonged in this line, we compared nuclei in the distal part (first half of the TZ, Video S18) to nuclei in the proximal part of the TZ (second half of the TZ, Video S19).

In the distal part of the TZ, we detected wild-type characteristics with respect to the number of aggregates (Figure S8A; Mann-Whitney test, *p* > 0.05), displacement tracks (Figure S7A and S7B), distances traveled (Figure S7D; Mann-Whitney test, *p* > 0.05), number of fusion/splitting events, and coalescence time (Figure S8B, S8C, S8D, S8E). In contrast, the distribution of the projected speed of SUN-1(S12E)::GFP was significantly reduced compared to the wild type (Figure S7C; Mann-Whitney test *p* < 0.05): the distribution between 40 and 160 nm/s was shifted towards the lower values and the ability of SUN-1(S12E)::GFP aggregates to reach a high projected speed (>160 nm/s) was reduced (4% long tails versus 10% long tails in the wild type). In contrast, in the proximal part of the prolonged TZ, SUN-1(S12E)::GFP aggregates showed a significant increase, both in their projected speed (16% long tails, Figure S7C′; Mann-Whitney test, *p* < 0.05) and the distance traveled, which reached values of 180° (Figure S7D′; Mann-Whitney test, *p* < 0.05). The average number of aggregates increased (Mann-Whitney test, *p* < 0.05). The number (Figure S8B and S8D) and periodicity (Figure S8C and S8E) of the fusion/splitting events remained in the wild-type range.

Phosphorylation of SUN-1 therefore affected the speed distribution and the distance traveled by the SUN-1 aggregates. Pairing was effective, but DSB repair was impaired in the SUN-1(S12E) mutant worms [28]. In other words, in the proximal part of the TZ, SUN-1 aggregates most likely represented paired homologs, which appeared to travel faster (16% long tails) than homologs in the process of pairing (distal part of the TZ; 4% long tails).

**SUN-1 patches tend to move faster than foci.**

SUN-1 aggregates are classified into foci and patches, and patches most likely represent many chromosome ends simultaneously assessing homology [1]. We followed the movement of SUN-1::GFP aggregates while keeping track of their size (Figure S9). This allowed us to address the question of whether the size of the aggregates has an influence on their instantaneous projected speed. A focus had a defined size of up to 2.15 µm^2^ and patches a size >2.15 µm^2^. We calculated the projected speed of foci and patches. No correlation between the size of the aggregates and their instantaneous projected speed was found (correlation factor for foci: −0.017 and for patches: −0.0169). The size of SUN-1 foci or patches, thus, had no influence on their respective speed.

However, there was a difference between foci and patches in their projected speed distribution. Both classes of aggregates were able to reach projected speeds higher than 160 nm/s (3% for the foci and 4% for the patches; Figure S9B), but their distribution was different (Mann-Whitney test, *p* < 0.05). Indeed, the cumulative distribution function (CDF) of the foci and the patches showed that foci tended to move more slowly than patches. From 16 to 120 nm/s, the CDF of the foci began to increase significantly compared to the CDF of SUN-1 patches (blue curve, Figure S9B). We conclude that although there was no direct link between the size of the aggregates and their projected speed, SUN-1 patches tended to move faster than SUN-1 foci.

1. Penkner A, Fridkin A, Gloggnitzer J, Baudrimont A, Machacek T, et al. (2009) Meiotic Chromosome Homology Search Involves Modifications of the Nuclear Envelope Protein Matefin/SUN-1. Cell 139: 920-933.
