# Supplementary material for: Leptotene/Zygotene Chromosome Movement Via the SUN/KASH Protein Bridge in Caenorhabditis elegans
Source: PLoS Genet. 2010 Nov 24;6(11):e1001219. doi: 10.1371/journal.pgen.1001219 (PMC2991264; doi:10.1371/journal.pgen.1001219)
Supplement: Text S2 — Supplemental methods. (0.03 MB DOC) [file pgen.1001219.s013.doc]

**Text S2. Supplemental methods.**

*Cytological preparation of gonads and immunostaining*

Hermaphrodite gonads were dissected and fixed as described in [2] for SUN-1 and ZIM-3 staining. Gonads were blocked in 1× PBS 3% BSA for 20 min after being fixed for one min in methanol (−20°C) and washed three times in 1× PBS 0.01% Tween-20. Antibodies were diluted in 1× PBS 0.01% sodium azide as follows: anti-GFP (catalog number 11814460001, Roche Diagnostics), 1:300 and anti-ZIM-3 [1], 1:100.

For SYP-1 staining, gonads were fixed as described in [3]. After dissection of gonads, samples were frozen in liquid nitrogen and then fixed for five min in methanol (−20°C). Twenty microliters of 3.7% formaldehyde was applied, and the samples were left at room temperature for 20 min. After washing three times in 1× PBS 0.01% Tween-20, antibodies raised against SYP-1 were applied at a dilution of 1:200 in 1× PBS 0.01% sodium azide.

1. Penkner A, Fridkin A, Gloggnitzer J, Baudrimont A, Machacek T, et al. (2009) Meiotic Chromosome Homology Search Involves Modifications of the Nuclear Envelope Protein Matefin/SUN-1. Cell 139: 920-933.
2. Martinez-Perez E, Villeneuve AM (2005) HTP-1-dependent constraints coordinate homolog pairing and synapsis and promote chiasma formation during C. elegans meiosis. Genes Dev 19: 2727-2743.
3. MacQueen AJ, Colaiacovo MP, McDonald K, Villeneuve AM (2002) Synapsis-dependent and -independent mechanisms stabilize homolog pairing during meiotic prophase in C. elegans. Genes Dev 16: 2428-2442.

*Fluorescence in situ hybridization*

5S ribosomal DNA (rDNA) was used as a probe for the right arm of chromosome V. 5S rDNA was labeled by PCR with digoxigenin-11-dUTP [4]. Digoxigenin-labeled probes were detected with FITC-conjugated anti-digoxigenin antibodies (1:100).

4. Pasierbek P, Jantsch M, Melcher M, Schleiffer A, Schweizer D, et al. (2001) A Caenorhabditis elegans cohesion protein with functions in meiotic chromosome pairing and disjunction. Genes Dev 15: 1349-1360.

*Microscopy and evaluation*

Evaluation of cytological phenotypes on fixed samples was performed on worms kept at 20°C for 18–24 h after preselecting them at the L4 stage. A Zeiss Axioskop epifluorescence microscope was used in combination with a cooled CCD camera (Photometrics) to take 3D stacks of images (MetaVue software; Universal Imaging, Downingtown, PA, USA). Stack pictures were deconvolved (AutoDeblur software; AutoQuant Imaging, Troy, NY, USA) and then projected (Helicon Focus software; http://helicon.com.ua/heliconfocus/). Artificial coloring and merging were done with Adobe Photoshop CS3 (Adobe Systems).
